# Differential accumulation of tau pathology between reciprocal F1 hybrids of rTg4510 mice

**DOI:** 10.1038/s41598-021-89142-2

**Published:** 2021-05-05

**Authors:** Daijiro Yanagisawa, Hamizah Shahirah Hamezah, Aslina Pahrudin Arrozi, Ikuo Tooyama

**Affiliations:** 1 Molecular Neuroscience Research Center, Shiga University of Medical Science, Seta Tsukinowa-Cho, Otsu, 520-2192 Japan; 2 Institute of Systems Biology (INBIOSIS), Universiti Kebangsaan Malaysia, 43600 UKM Bangi, Selangor Malaysia

**Keywords:** Neurodegenerative diseases, Alzheimer's disease

## Abstract

Tau, a family of microtubule-associated proteins, forms abnormal intracellular inclusions, so-called tau pathology, in a range of neurodegenerative diseases collectively known as tauopathies. The rTg4510 mouse model is a well-characterized bitransgenic F1 hybrid mouse model of tauopathy, which was obtained by crossing a Camk2α-tTA mouse line (on a C57BL/6 J background) with a tetO-MAPT*P301L mouse line (on a FVB/NJ background). The aim of this study was to investigate the effects of the genetic background and sex on the accumulation of tau pathology in reciprocal F1 hybrids of rTg4510 mice, i.e., rTg4510 on the (C57BL/6 J × FVB/NJ)F1 background (rTg4510_CxF) and on the (FVB/NJ × C57BL/6 J)F1 background (rTg4510_FxC). As compared with rTg4510_CxF mice, the rTg4510_FxC mice showed marked levels of tau pathology in the forebrain. Biochemical analyses indicated that the accumulation of abnormal tau species was accelerated in rTg4510_FxC mice. There were strong effects of the genetic background on the differential accumulation of tau pathology in rTg4510 mice, while sex had no apparent effect. Interestingly, *midline-1* (*Mid1*) was identified as a candidate gene associated with this difference and exhibited significant up/downregulation according to the genetic background. *Mid1* silencing with siRNA induced pathological phosphorylation of tau in HEK293T cells that stably expressed human tau with the P301L mutation, suggesting the role of *Mid1* in pathological alterations of tau. Elucidation of the underlying mechanisms will provide novel insights into the accumulation of tau pathology and is expected to be especially informative to researchers for the continued development of therapeutic interventions for tauopathies.

## Introduction

Tau belongs to a family of microtubule-associated proteins and was originally identified as a heat-stable protein essential for microtubule assembly^1^. Human tau is encoded by the *MAPT* gene on chromosome 17q21^2^ and consists of six isoforms, 0N3R, 1N3R, 2N3R, 0N4R, 1N4R, and 2N4R, which are generated by alternative splicing of exon 2 (E2), E3, and E10^3–5^.

Tau forms abnormal intracellular inclusions, so-called tau pathology, in neurons and glial cells in a range of neurodegenerative diseases collectively known as tauopathies, which include Alzheimer’s disease, progressive supranuclear palsy, corticobasal degeneration, and Pick’s disease. Tau pathology is characterized by abnormally hyperphosphorylated tau molecules that form bundles of paired helical filaments and straight filaments^6,7^. In 1998, missense mutations of tau were identified in patients with frontotemporal dementia associated with parkinsonism linked to chromosome 17^8–10^. This discovery demonstrated that the pathological abnormalities of tau can be a primary or even the sole cause of tauopathy^7^.

rTg4510 is a well-characterized bitransgenic F1 hybrid mouse model of tauopathy. rTg4510 mice were originally produced by crossing a responder transgenic mouse (tetO-MAPT*P301L mouse line on a FVB/NJ background) with an activator transgenic mouse (Camk2α-tTA mouse line on a 129S6 background). The responder carries a tetracycline response element placed upstream in a complementary DNA (cDNA) encoding human 0N4R tau with a P301L mutation that is linked to hereditary tauopathy, while the activator expresses a tetracycline-controlled transactivator (tTA) under the control of the Ca^2+^-calmodulin kinase II α (Camk2α) promoter^11,12^. Consequently, the hybrid F1 mice express human P301L mutant tau in the forebrain at a level approximately 13-fold higher than that of endogenous mouse tau and display progressive age-dependent accumulation of tau pathology, which can include abnormal tau phosphorylation and argyrophilic inclusions in the forebrain region, decreased dendritic spine density, neurodegeneration, and a variety of cognitive deficits^11–17^.

In a recent study, the original 129S6 background of the Camk2α-tTA mouse line was replaced with the C57BL/6 background, one of the most commonly used mouse strains, and reported that the introduction of the C57BL/6 background into the rTg4510 mouse background altered the presentation of tau pathology only minimally from the original phenotype and with no loss of fidelity^18^. In contrast, we previously reported a significant difference in the accumulation of tau pathology in male reciprocal F1 hybrids of rTg4510 mice^19^. In addition, Yue et al. reported a sex difference in tau pathology and memory decline in the original rTg4510 mice^20^. The importance of the genetic background and sex in the phenotype of transgenic mouse model is well known. Therefore, in the present study, the mechanisms underlying differential tau accumulation in rTg4510 mice were investigated, while focusing on the effects of the genetic background and sex. A deeper understanding of tau pathology will be useful for the development of therapeutic regimens for tauopathies.

## Results

### Differential accumulation of tau pathology between reciprocal F1 hybrids

**Figure 1:**
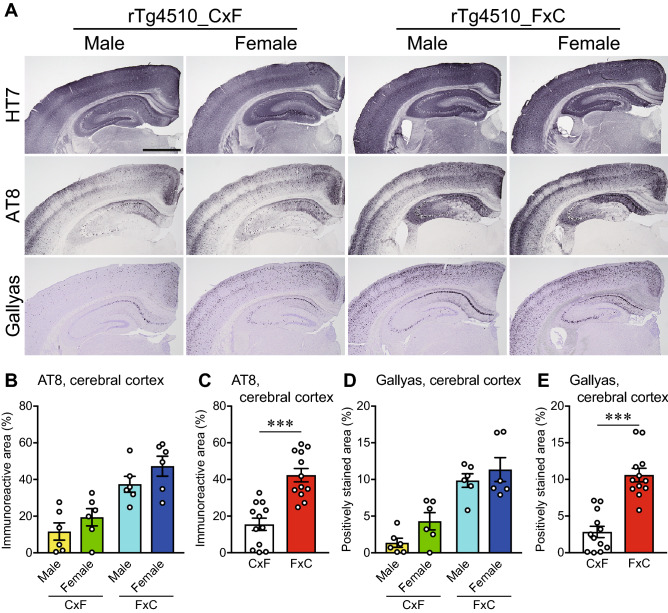
Abnormally phosphorylated tau and Gallyas-positive structures in the brains of rTg4510 mice at 6 months of age. (**A**) Representative photographs of the immunohistochemical results for human tau (clone HT7), phosphorylated tau (clone AT8), and Gallyas silver staining. Scale bar: 1 mm. (**B**–**E**) Quantitative analysis of the AT8-immunoreactive area and Gallyas-positive stained area. Two-way ANOVA (genetic background × sex) revealed a main effect of the genetic background, but no effect of sex, in the AT8-immunoreactive area (**B**) and Gallyas-positive stained area (**D**) of the cerebral cortex (Supplementary Table S1). The Mann–Whitney *U* test revealed significantly higher levels of AT8-immunoreactivity (**C**) and Gallyas-positive staining (**E**) in samples obtained from the rTg4510_FxC mice, as compared with the rTg4510_CxF mice. The rTg4510_CxF and rTg4510_FxC groups contained six males and six females each (*n* = 12/group). Data are presented as the mean ± SEM. ***p < 0.001 (Mann–Whitney *U* test).

Reciprocal F1 hybrids of rTg4510 mice consisting of two genetic backgrounds (Table 1) were used in this study: rTg4510 mice on a (C57BL/6 J × FVB/NJ)F1 (maternal strain × paternal strain) background (rTg4510_CxF) and rTg4510 mice on a (FVB/NJ × C57BL/6 J)F1 background (rTg4510_FxC). To investigate the accumulation of tau pathology, histological analysis was performed of coronal brain sections from male and female rTg4510_CxF and rTg4510_FxC mice at 6 months of age. Immunohistochemical analysis of human tau (HT7) revealed a high level of immunoreactivity in the cerebral cortex and hippocampus in all groups although there were no significant differences (Fig. 1A). Immunohistochemical analysis of phosphorylated tau (AT8) and Gallyas silver staining unveiled a large number of positively stained structures in the cerebral cortex and hippocampus of rTg4510 mice (Fig. 1A). Quantitative analysis was performed of the AT8-immunoreactive areas and Gallyas-positive stained areas of the cerebral cortex of rTg4510 mice. Two-way ANOVA with the genetic background and sex as factors revealed that the genetic background had the most profound effect [F (1, 20) = 31.18, *p* < 0.0001], while sex had no effect [F (1, 20) = 3.37, *p* = 0.0813] in the AT8-immunoreactive areas of the cerebral cortex (Fig. 1B). Subsequent analysis with the Mann–Whitney *U* test indicated significantly greater AT8-immunoreactivity in rTg4510_FxC mice than rTg4510_CxF mice (*p* < 0.0001; Fig. 1C). Furthermore, two-way ANOVA with the genetic background and sex as factors revealed that the genetic background had the most profound effect [F (1, 20) = 45.74, *p* < 0.0001], while sex had no effect [F (1, 20) = 3.726, *p* = 0.0679] in the Gallyas-positive stained areas of the cerebral cortex (Fig. 1D). Gallyas-positive staining of rTg4510_FxC mice was significantly greater than that of rTg4510_CxF mice (*p* < 0.0001; Fig. 1E). Quantitative analysis also revealed that the genetic background, but not sex, had a significant main effect in the AT8-immunoreactive areas and Gallyas-positive stained areas of the hippocampus, and significantly higher levels of these areas in rTg4510_FxC mice, as compared with rTg4510_CxF mice (Supplementary Fig. S1, Table S1). These results suggest more severe accumulation of tau pathology in rTg4510_FxC mice than rTg4510_CxF mice. In addition, there was no significant sex difference in the accumulation of tau pathology detected in rTg4510 mice.

**Table 1:** The genetic backgrounds of the two mouse strains obtained by reciprocal crosses.

Offspring	Genetic background (maternal strain × paternal strain)F1	Parents	
		Maternal strain	Paternal strain
CxF	(C57BL/6 J × FVB/NJ)F1	C57BL/6 J (Hemizygous Camk2α-tTA)	FVB/NJ (Hemizygous tetO-MAPT*P301L)
FxC	(FVB/NJ × C57BL/6 J)F1	FVB/NJ (Hemizygous tetO-MAPT*P301L)	C57BL/6 J (Hemizygous Camk2α-tTA)

### Soluble and insoluble tau accumulation differs according to the genetic background

**Figure 2:**
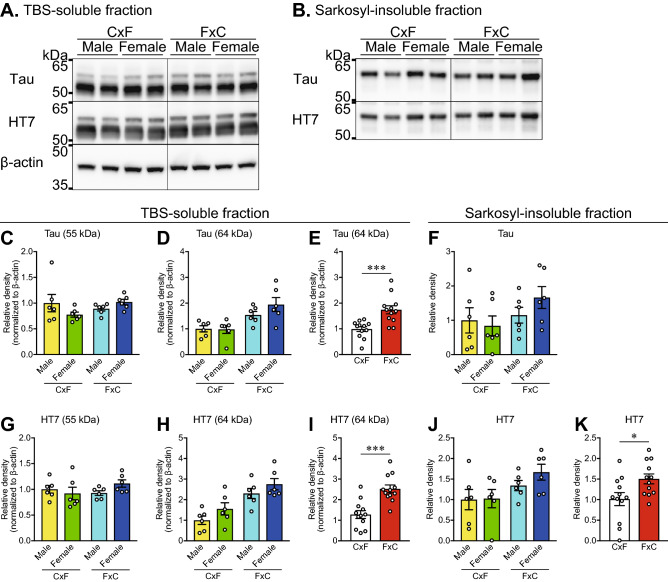
Accumulation of soluble and insoluble tau in rTg4510 mice at 6 months of age. (**A**,**B**) Representative images of western blots for Tau and HT7 in the TBS-soluble fraction (**A**) and sarkosyl-insoluble fraction (**B**) of the cerebral cortex. β-actin was used as a loading control. The band images in each row were obtained from the same gel with separation of the CxF and FxC samples. Full-length blots are presented in Supplementary Figure S11. (**C**–**K**) Densitometric analysis of tau accumulation. Two-way ANOVA (genetic background × sex) revealed a main effect of the genetic background, but no effect of sex, on levels of Tau (**D**) and HT7 (**H**) at 64 kDa in the TBS-soluble fraction and HT7 (**J**) in the sarkosyl-insoluble fraction (Supplementary Table S1). The Mann–Whitney U test revealed significantly higher levels of Tau (**E**) and HT7 (**I**) at 64 kDa in the TBS-soluble fraction and HT7 (**K**) in the sarkosyl-insoluble fraction from rTg4510_FxC mice, as compared with rTg4510_CxF mice. The rTg4510_CxF and rTg4510_FxC groups contained six males and six females each (n = 12/group). Data are presented as the mean ± SEM. ***p < 0.001, *p < 0.05 (Mann–Whitney *U* test).

Western blot analysis of the TBS-soluble and sarkosyl-insoluble fractions extracted from the cerebral cortex of rTg4510 mice was performed to investigate the accumulation of soluble and insoluble tau in the brain. With the use of polyclonal (Tau) and monoclonal (HT7) antibodies, Tau was detected at 55 kDa as a major band and at around 64 kDa as a minor band in the TBS-soluble fraction (Fig. 2A); however, sarkosyl-insoluble tau was only detected at around 64 kDa (Fig. 2B). Two-way ANOVA (genetic background × sex) of the densitometric analysis results revealed no effect of the genetic background [F (1, 20) = 0.495, *p* = 0.4898 for Tau, F (1, 20) = 0.5535, *p* = 0.4656 for HT7] or sex [F (1, 20) = 0.2426, *p* = 0.6277 for Tau, F (1, 20) = 0.3885, *p* = 0.5401 for HT7] on 55-kDa tau levels in the TBS-soluble fraction (Fig. 2C,G). In contrast, two-way ANOVA (genetic background × sex) revealed a significant main effect of the genetic background [F (1, 20) = 17.12, *p* = 0.0005 for Tau, F (1, 20) = 23.49, *p* < 0.0001 for HT7], but not sex [F (1, 20) = 1.118, *p* = 0.3030 for Tau, F (1, 20) = 3.783, *p* = 0.0660 for HT7], on 64-kDa tau levels in the TBS-soluble fraction (Fig. 2D,H). The level of 64-kDa tau in the TBS-soluble fraction was significantly higher in rTg4510_FxC mice than rTg4510_CxF mice (*p* = 0.0005 for Tau, *p* = 0.0003 for HT7; Fig. 2E,I). In the sarkosyl-insoluble fraction, the genetic background and sex had no effect on 64-kDa tau levels probed with the polyclonal antibody (Tau), as determined by two-way ANOVA (Fig. 2F). In contrast, two-way ANOVA (genetic background × sex) revealed that the genetic background had a significant main effect [F (1, 20) = 5.937, *p* = 0.0243], but not sex [F (1, 20) = 0.7476, *p* = 0.3975], on sarkosyl-insoluble tau levels probed with the HT7 antibody (Fig. 2J). The level of sarkosyl-insoluble tau probed with HT7 was significantly higher in rTg4510_FxC mice than rTg4510_CxF mice (*p* = 0.0197; Fig. 2K). These results suggest greater accumulation of abnormal tau species detected at around 64 kDa in rTg4510_FxC mice, as compared with rTg4510_CxF mice, although there was no significant difference between groups in normal tau protein levels, which are generally measured in soluble protein extracts at 55 kDa.

### Effect of genetic background on the abnormal phosphorylation of tau

**Figure 3:**
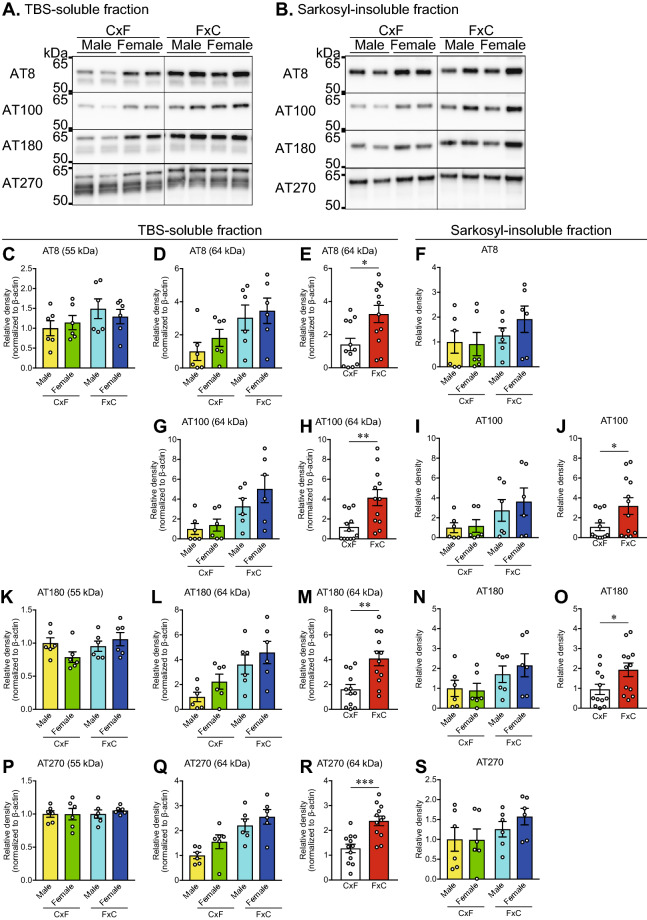
Accumulation of abnormally phosphorylated tau in rTg4510 mice at 6 months of age. (**A**,**B**) Representative images of western blots of phosphorylated tau (AT8, AT100, AT180, and AT270) in the TBS-soluble fraction (**A**) and sarkosyl-insoluble fraction (**B**) from the cerebral cortex. The band images of each row were obtained from same gel with separation of the CxF and FxC samples. Full-length blots are presented in Supplementary Figure S12. (**C**–**S**) Densitometric analysis of phosphorylated tau. Two-way ANOVA (genetic background × sex) revealed a main effect of the genetic background, but no effect of sex, on the levels of AT8 (**D**), AT100 (**G**), AT180 (**L**), and AT270 (**Q**) at 64 kDa in the TBS-soluble fraction, and AT100 (**I**) and AT180 (**N**) in the sarkosyl-insoluble fraction (Supplementary Table S1). The Mann–Whitney *U* test revealed significantly higher levels of AT8 (**E**), AT100 (**H**), AT180 (**M**), and AT270 (**R**) at 64 kDa in the TBS-soluble fraction and AT100 (**J**) and AT180 (**O**) in the sarkosyl-insoluble fraction of the rTg4510_FxC mice, as compared with the rTg4510_FxC mice. The rTg4510_CxF and rTg4510_FxC groups contained six males and six females each (*n* = 12/group). Data are presented as mean ± SEM. ***p < 0.001, **p < 0.01, *p < 0.05 (Mann–Whitney *U* test).

Subsequently, western blot analysis of abnormally phosphorylated tau in the TBS-soluble and sarkosyl-insoluble fractions was performed. Phosphorylated tau probed with AT8 (pSer202 and pThr205), AT180 (pThr231), and AT270 (pThr181) in the TBS-soluble fraction was revealed as a major band at 64-kDa and a minor band at 55-kDa, while AT100 (pThr212 and pSer214) showed only a 64-kDa band (Fig. 3A). In the sarkosyl-insoluble fraction, phosphorylated tau probed with AT8, AT100, AT180, and AT270 was only detected at around 64 kDa (Fig. 3B). Following densitometric analysis of the TBS-soluble fraction, two-way ANOVA (genetic background × sex) revealed no effects of the genetic background [F (1, 20) = 2.538, *p* = 0.1268 for AT8, F (1, 20) = 1.757, *p* = 0.2000 for AT180, F (1, 20) = 0.2224, *p* = 0.6424 for AT270] or sex [F (1, 20) = 0.01865, *p* = 0.8927 for AT8, F (1, 20) = 0.3825, *p* = 0.5432 for AT180, F (1, 20) = 0.197, *p* = 0.6619 for AT270] on levels of 55-kDa phosphorylated tau (Fig. 3C,K,P). In contrast, two-way ANOVA (genetic background × sex) revealed a significant main effect of the genetic background [F (1, 20) = 7.854, *p* = 0.0110 for AT8, F (1, 20) = 10.81, *p* = 0.0037 for AT100, F (1, 20) = 12.75, *p* = 0.0019 for AT180, F (1, 20) = 19.72, *p* = 0.0003 for AT270], but not sex [F (1, 20) = 0.8822, *p* = 0.3588 for AT8, F (1, 20) = 1.425, *p* = 0.2466 for AT100, F (1, 20) = 2.527, *p* = 0.1276 for AT180, F (1, 20) = 3.219, *p* = 0.0879 for AT270], on levels of 64-kDa phosphorylated tau in the TBS-soluble fraction (Fig. 3D,G,L,Q). The levels of 64-kDa phosphorylated tau in the TBS-soluble fraction were significantly higher in rTg4510_FxC mice than rTg4510_CxF mice (*p* = 0.0121 for AT8, *p* = 0.0029 for AT100, *p* = 0.0045 for AT180, *p* = 0.0004 for AT270; Fig. 3E,H,M,R). Probing of phosphorylated tau in the sarkosyl-insoluble fraction with AT100 and AT180 revealed a significant main effect of the genetic background [F (1, 20) = 4.72, *p* = 0.0420 for AT100, F (1, 20) = 4.868, *p* = 0.0392 for AT180], but not sex [F (1, 20) = 0.3025, *p* = 0.5884 for AT100, F (1, 20) = 0.1474, *p* = 0.7051 for AT180], as determined by two-way analysis of variance (ANOVA) (Fig. 3I,N), while the genetic background and sex had no effect on phosphorylated tau in the sarkosyl-insoluble fraction with the use of the AT8 and AT270 probes (Fig. 3F,S). Probing of the sarkosyl-insoluble fraction with AT100 and AT180 revealed significantly higher levels of phosphorylated in rTg4510_FxC mice than rTg4510_CxF mice (*p* = 0.0377 for AT100, *p* = 0.0284 for AT180; Fig. 3J,O). We further analyzed the ratios of phosphorylated tau to total tau which were detected at 55 and 64 kDa in the TBS-soluble fraction and at 64 kDa in the sarkosyl-insoluble fraction (Supplementary Fig. S2). There were no significant changes in the ratio of phosphorylated tau to total tau, which was detected at 55 kDa in the TBS-soluble fraction (Supplementary Fig. S2). A two-way ANOVA (genetic background × sex) revealed an effect of genetic background, but no effect of sex on the ratios of AT8 [F (1, 20) = 6.813, p = 0.0168 for genetic background, F (1, 20) = 2.257, p = 0.1486 for sex], AT100 [F (1, 20) = 8.999, p = 0.0071 for genetic background, F (1, 20) = 0.7756, p = 0.3889 for sex], and AT180 [F (1, 20) = 6.853, p = 0.0184 for genetic background, F (1, 20) = 2.11, p = 0.1619 for sex], but not AT270 [F (1, 20) = 1.06, p = 0.3155 for genetic background, F (1, 20) = 1.506, p = 0.2340 for sex], to total tau, which was detected at 64 kDa in the TBS-soluble fraction. The Mann–Whitney U test revealed significantly higher ratios of AT8, AT100, and AT180 to total tau in rTg4510_FxC mice compared with rTg4510_FxC mice (p = 0.0100 for AT8, p = 0.0055 for AT100, p = 0.0242 for AT180; Supplementary Fig. S2). A two-way ANOVA of the ratios of AT100 and AT180 to total tau in the sarkosyl-insoluble fraction primarily revealed effects of genetic background [F (1, 20) = 5.425, p = 0.0304 for AT100, F (1, 20) = 10.6, p = 0.0040 for AT180], but no effect of sex [F (1, 20) = 0.02554, p = 0.8746 for AT100, F (1, 20) = 0.4481, p = 0.5109 for AT180] in rTg4510 mice. The Mann–Whitney U test revealed significantly higher ratios of AT100 and AT180 to total tau in rTg4510_FxC mice compared with rTg4510_FxC mice (p = 0.0464 for AT8, p = 0.0038 for AT100; Supplementary Fig. S2). Taken together, these results suggest that the genetic background of rTg4510 mice may affect not only the increase in the levels of abnormal tau, but also the increase in the ratio of tau phosphorylation, in particular at the AT100 and AT180 sites, and in part at the AT8 site.

### No difference in tau mRNA levels between reciprocal F1 hybrids

**Figure 4:**
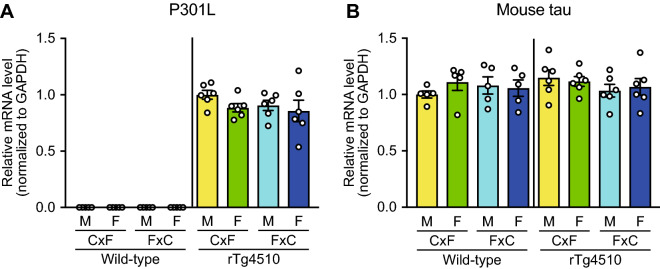
The mRNA levels of human P301L tau and mouse tau in rTg4510 mice at 6 months of age. qPCR analyses of transgenic human P301L tau (**A**) and endogenous mouse tau (**B**) in WT and rTg4510 mice were conducted. Two-way ANOVA (genetic background × sex) revealed no effects of the genetic background or sex on the mRNA levels of transgenic human P301L tau and endogenous mouse tau (Supplementary Table S2). The WT_CxF and WT_FxC groups contained four males and four females each (*n* = 8/group). The rTg4510_CxF and rTg4510_FxC groups contained six males and six females each (*n* = 12/group). Data are presented as the mean ± SEM.

Next, the effects of the genetic background on tau mRNA levels were investigated. The mRNA levels of transgenic human P301L tau, which were undetectable in wild-type (WT) mice on a (C57BL/6 J × FVB/NJ)F1 background (WT_CxF) and on a (FVB/NJ × C57BL/6 J)F1 background (WT_FxC), had no effect on the genetic background or sex of rTg4510 mice [two-way ANOVA (genetic background × sex); F (1, 20) = 1.086 *p* = 0.3099 for genetic background, F (1, 20) = 1.916, *p* = 0.1816 for sex; Fig. 4A]. The mRNA levels of mouse tau were similar in rTg4510 and WT mice. Two-way ANOVA (genetic background × sex) revealed that the genetic background and sex had no effect on mouse tau mRNA levels in the WT mice [F (1, 16) = 0.07445, *p* = 0.7885 for genetic background, F (1, 16) = 0.09461, *p* = 0.7624 for sex] or rTg4510 mice [F (1, 20) = 1.765, *p* = 0.1990 for genetic background, F (1, 20) = 2.929e-005, *p* = 0.9957 for sex; Fig. 4B]. These results suggest that the mouse tau mRNA levels were not affected by the genetic background.

### Identification of a gene related to the differential accumulation of tau in rTg4510 mice

We attempted to identify genes related to the difference in tau accumulation between rTg4510_CxF and rTg4510_FxC mice. Transgene insertion has been reported to cause a 244-kb deletion on chromosome 14 of tetO-MAPT*P301L mice, which affects *Fgf14* and a 508-kbp deletion on chromosome 12 of Camk2α-tTA mice, which affects *Vipr2*, *Wdr60*, *Esyt2*, *D430020J02Rik*, and *Ncapg2*^21^. We therefore attempted to determine if the genetic background had any effect of the mRNA levels of particular genes in WT and rTg4510 mice. However, the results revealed no significant effects of the genetic background on the mRNA levels of the tested genes between reciprocal F1 hybrids (Supplementary Fig. S3, Table S2, S3). Subsequently, we investigated whether there were differences in the mRNA levels of disease-associated tau kinases and select phosphatases, namely cyclin-dependent-like kinase 5, dual specificity tyrosine-phosphorylation-regulated kinase 1A isoform 1, glycogen synthase kinase-3 β, casein kinase 1 alpha 1, microtubule affinity-regulating kinase 1 (*Mark1*), *Mark2, Mark3, Mark4*, protein kinase cAMP-activated catalytic subunit alpha, and protein phosphatase 2 catalytic subunit alpha. However, the genetic background and sex had no effect of on the mRNA levels of these genes (Supplementary Fig. S4, Table S2, S3).

**Figure 5:**
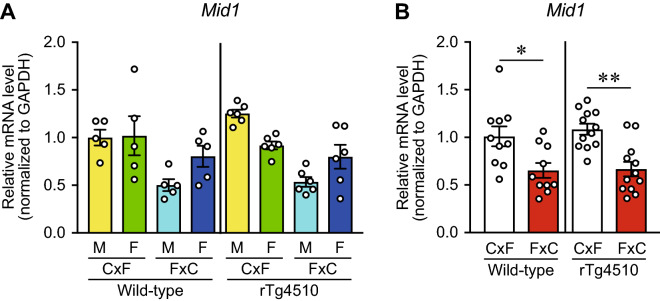
The *Mid1* gene may be related to differential accumulation of tau. (**A**) Two-way ANOVA (genetic background × sex) of the qPCR results revealed a main effect of the genetic background, but no effect of sex, on the mRNA levels of *Mid1* in WT and rTg4510 mice (Supplementary Table S3). (**B**) The Mann–Whitney *U* test revealed significantly higher *Mid1* mRNA levels in WT_CxF than in WT_FxC mice and in rTg4510_CxF as compared with rTg4510_FxC mice. The WT_CxF and WT_FxC groups contained five males and five females each (*n* = 10/group). The rTg4510_CxF and rTg4510_FxC groups contained six males and six females each (*n* = 12/group). Data are presented as the mean ± SEM. **p < 0.01, *p < 0.05 (Mann–Whitney *U* test).

Next, qPCR analysis of 10 upregulated and downregulated genes was performed (Fig. 4, Supplementary Fig. S5, S6, Table S4), based on the RNA-seq analysis results of male WT_CxF (*n* = 1) and male WT_FxC (*n* = 1) mice. The results revealed a significant difference in the mRNA levels of *midline-1 (Mid1)* between reciprocal F1 hybrids. Two-way ANOVA (genetic background × sex) revealed that the genetic background, but not sex, had a significant effect on *Mid1* levels in WT mice [F (1, 16) = 7.832, *p* = 0.0129 for genetic background, F (1, 16) = 1.577, *p* = 0.2273 for sex] and rTg4510 mice [F (1, 20) = 32.46, *p* < 0.0001 for genetic background, F (1, 20) = 0.2321, *p* = 0.6352 for sex; Fig. 5A]. The levels of *Mid1* in WT_CxF and rTg4510_CxF mice were significantly higher than those in WT_FxC and rTg4510_FxC mice, respectively (*p* = 0.0122 for WT, *p* = 0.0007 for rTg4510; Fig. 5B).

### Tau accumulation and *Mid1* mRNA levels in rTg4510 mice at 3 months of age

Histological and western blot analyses of male and female rTg4510_CxF and rTg4510_FxC mice at 3 months of age were performed. Histological analysis showed no significant differences in AT8-immunoreactivity and Gallyas-positive staining between rTg4510_CxF and rTg4510_FxC mice (Supplementary Fig. S7). Western blot analysis of the TBS-soluble fraction showed significant differences in AT8 levels, but not levels of HT7, AT180, and AT270 (Supplementary Fig. S8A-K). By two-way ANOVA (genetic background × sex), there was a main effect of the genetic background, but not sex, on AT8 levels at 55 kDa [F (1, 12) = 5.919, *p* = 0.0316 for genetic background, F (1, 12) = 0.5805, *p* = 0.4608 for sex] and 64 kDa [F (1, 12) = 12.9, *p* = 0.0037 for genetic background, F (1, 12) = 0.8891, *p* = 0.3643 for sex; Supplementary Fig. S8D and E], while the Mann–Whitney *U* test revealed significantly high levels of AT8 at 55 kDa (*p* = 0.0207) and 64 kDa (*p* = 0.0207) in rTg4510_FxC mice, as compared with rTg4510_CxF mice (Supplementary Fig. S8F and G). In contrast, two-way ANOVA (genetic background × sex) revealed no effects of the genetic background or sex on the levels of HT7, AT8, AT100, AT180, and AT270 in the sarkosyl-insoluble fractions between the rTg4510_CxF and rTg4510_FxC mice (Supplementary Fig. S8L-Q).

The genetic background, but not sex, had an effect on *Mid1* mRNA levels in rTg4510 mice at 3 months of age, as determined by two-way ANOVA (genetic background × sex) [F (1, 12) = 6.733, *p* = 0.0234 for genetic background, F (1, 12) = 0.6322, *p* = 0.4420 for sex; Supplementary Fig. S9A]. The Mann–Whitney *U* test revealed significantly lower *Mid1* mRNA levels in rTg4510_FxC mice, as compared with rTg4510_CxF mice (*p* = 0.0095; Supplementary Fig. S9B).

### *MID1* silencing induced pathological phosphorylation of tau

**Figure 6:**
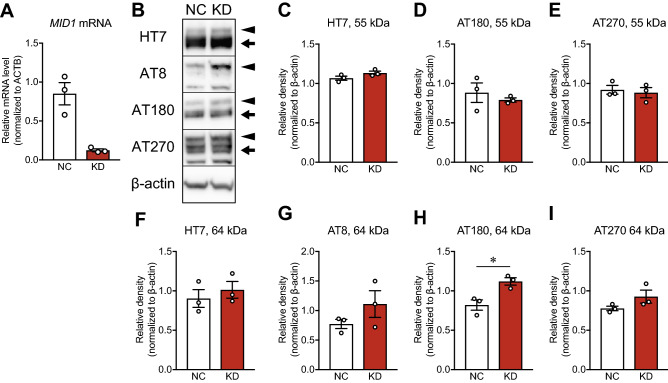
The level of tau accumulation and phosphorylation in *MID1*-knockdown cells. HEK293T cells stably expressing human tau with the P301L mutation were treated with negative control siRNA (NC) and *MID1* siRNA (KD) for 48 h. (**A**) *MID1* mRNA levels in NC-treated and *MID1*-knockdown cells. (**B**) Western blots of HT7, AT8, AT180, AT270, and β-actin in the TBS-soluble fractions. Arrows and arrowheads indicate bands at 55 and 64 kDa, respectively. Full-length blots are presented in Supplementary Figure S13. (**C**–**I**) Densitometric analysis. Data are presented as the mean ± SEM of three independent experiments. *p < 0.05 (Student’s *t*-test).

To investigate the role of *Mid1* in tau phosphorylation and accumulation, *MID1* silencing was performed with siRNA in HEK293T cells stably expressing human tau with the P301L mutation. The qPCR results showed that *MID1* mRNA levels were reduced by 88% in *MID1*-knockdown cells, as compared with control cells (Fig. 6A). Western blot analysis of the TBS-soluble fraction showed no significant differences in the levels of HT7, AT180, and AT270 at 55 kDa between cells treated with negative control siRNA and *MID1* siRNA (Fig. 6B–E). *MID1*-knockdown cells showed a significant increase in AT180 levels at 64 kDa (*p* = 0.0206, Student’s *t*-test) and slight, but not significant, increases in the levels of HT7, AT8 and AT270 at 64 kDa (Fig. 6F–I). Western blot analysis of the sarkosyl-insoluble fractions of *MID1*-knockdown cells was also performed; however, no bands were detectable with the use of the HT7, AT8, AT180, and AT270 antibodies (data not shown). We further analyzed the ratios of phosphorylated tau to total tau; however, there were no significant differences in these ratios between cells treated with negative control siRNA and *MID1* siRNA (Supplementary Fig. S10).

### No relationship of the number of siblings, body weight, and brain weight with differential tau pathology

We finally investigated whether the genetic background resulted in changes to the number of siblings, body weight, and brain weight (Table 2). Two-way ANOVA revealed no significant effect of the genetic background or sex on the number of siblings in WT mice [F (1, 24) = 1.607, *p* = 0.2171 for genetic background, F (1, 24) = 0.1785, *p* = 0.6764 for sex] or rTg4510 mice [F (1, 32) = 0.7536, *p* = 0.3918 for genetic background, F (1, 32) = 0.3612, *p* = 0.5521 for sex]. However, the genetic background and sex had significant effects on the body weight of WT mice [F (1, 24) = 20.02, *p* = 0.0002 for genetic background, F (1, 24) = 10.43, *p* = 0.0036 for sex], while sex, but not the genetic background, had an effect the body weight of rTg4510 mice [F (1, 32) = 1.432, *p* = 0.2402 for genetic background, F (1, 32) = 34.68, *p* < 0.0001 for sex]. Furthermore, sex, but not the genetic background, had an effect on the wet weight of the cerebral cortex in WT mice [F (1, 16) = 4.268, *p* = 0.0554 for genetic background, F (1, 16) = 5.151, *p* = 0.0374 for sex], but not in rTg4510 mice [F (1, 20) = 3.181, *p* = 0.0897 for genetic background, F (1, 20) = 0.678, *p* = 0.4200 for sex]. Based on these results, the number of siblings, body weight, and brain weight appear to have no effect differential tau accumulation in rTg4510 mice.

**Table 2:** Summary of the number of siblings, body weights, and brain weights.

Mouse	Sex	No. of siblings	Body weight (g)	Wet weight of the cerebral cortex (mg)
WT_CxF	Male	7.4 ± 0.8	29.5 ± 0.5	100.1 ± 4.3
	Female	7.1 ± 0.8	26.4 ± 0.5	108.0 ± 4.4
WT_FxC	Male	8.9 ± 0.9	35.9 ± 1.5	107.1 ± 3.2
	Female	8.3 ± 1.4	31.0 ± 1.8	117.5 ± 4.1
rTg4510_CxF	Male	6.8 ± 0.8	27.0 ± 0.6	79.1 ± 4.8
	Female	7.6 ± 0.3	22.9 ± 0.6	79.7 ± 3.2
rTg4510_FxC	Male	7.8 ± 0.9	28.7 ± 0.8	75.7 ± 4.1
	Female	8.0 ± 1.1	23.1 ± 1.2	68.4 ± 4.1

## Discussion

We investigated tau pathology in reciprocal F1 hybrids of rTg4510 mice at 3 and 6 months of age. The results showed more severe pathological tau accumulation and phosphorylation in rTg4510_FxC mice than rTg4510_CxF mice with no obvious sex difference at 6 month of age. In contrast, there were no obvious effects of the genetic background or sex on pathological alterations of tau in rTg4510 mice at 3 months of age. There was, however, a significant difference in *Mid1* mRNA levels between the reciprocal F1 hybrids, and *MID1* silencing with siRNA induced pathological phosphorylation of tau in HEK293T cells stably expressing human tau with the P301L mutation. These findings suggest that downregulation of Mid1 contributes to severe pathological alterations of tau in rTg4510_FxC mice, although the effect might be somewhat weak and will require a considerable amount of time to be apparent in the in vivo brain.

The *Mid1* gene is located on the X chromosome and encodes a 667-amino acid protein that belongs to the tripartite motif family and consists of a RING finger domain, two B-box domains, a coiled-coil region, a fibronectin type III domain, and B30.2 domains. The Mid1 protein has been shown to function as an E3 ubiquitin ligase targeting the microtubule-associated catalytic subunit of protein phosphatase 2A (PP2Ac) in ubiquitin-mediated degradation^22^. The Mid1 protein is, however, involved in signaling of mammalian target of rapamycin complex 1 through the regulation of PP2A activity^23^. PP2A is a phosphatase that targets tau and mTOR signaling is known to regulate tau phosphorylation and degradation^24^. Considering the previous studies cited above, Mid1 upregulation would be expected to increase tau phosphorylation and accumulation through the inhibition of PP2A degradation and the activation of mTOR, which contradicts the results of the present study. Therefore, it is of interest to investigate the difference in the level/activity of PP2A between reciprocal F1 hybrids of rTg4510 mice in future studies. However, it is difficult to predict the role of Mid1 in the accumulation of tau pathology, since it has been reported to serve other functions, such as the regulation of mRNA translation by direct association with the mRNA^25^. Furthermore, mutations to the *Mid1* gene have been identified in patients with X-linked Opitz G/BBB syndrome, an inherited multiple-organ disorder primarily affecting midline structures^26,27^, although the pathological mechanisms remain unknown. Taken together, although the role of Mid1 in pathological alterations of tau in the in vivo brain remains to be elucidated, the results of the present study provide novel insight into the mechanism underlying the pathological alterations of tau in tauopathy.

Although the mechanisms underlying the difference in *Mid1* levels between mice with the (C57BL/6 J × FVB/NJ)F1 and (FVB/NJ × C57BL/6 J)F1 backgrounds are currently unclear, we expect that epigenetic mechanisms are involved, as it has been proposed that maternal care and environmental factors (e.g., diet, stress, etc.) may influence epigenetic changes in offspring^28,29^. Mice on the (C57BL/6 J × FVB/NJ)F1 background are delivered from mothers on a C57BL/6 J background, while mice on the (FVB/NJ × C57BL/6 J)F1 background are delivered from mothers on a FVB/NJ background. Therefore, maternal strain-dependent epigenetic changes may occur in offspring between the prenatal period and weaning; consequently, expression levels of genes, including Mid1, would differ depending on the maternal strain.

Mitochondrial DNA (mtDNA) has also been suspected in the differential accumulation of tau, because of its maternal inheritance. A previous study reported variations in mtDNA between strains C57BL/6 J and FVB/NJ^30,31^. Specifically, the authors reported a G to T substitution at position 7778, which results in an amino acid substitution (aspartic acid to tyrosine) in adenosine triphosphate synthase 8. In addition, a T to C substitution at position 9461 resulted in no amino acid change (methionine to methionine) in NADH dehydrogenase 3. Furthermore, polymorphic variants contribute to some phenotypic differences in mice^32,33^. Mitochondria are the powerhouses of the cell, generating cellular energy in the form of ATP through oxidative phosphorylation (OXPHOS)^34^. Mammalian mtDNA encodes 13 polypeptides that are subunits of OXPHOS complexes, 22 transfer RNAs, and two ribosomal RNAs that are necessary for the translation of 13 polypeptides. Hence, mtDNA mutations affect energy production via the OXPHOS system^34–36^. Since maintaining electrochemical gradients and releasing and recycling synaptic vesicles are highly energy-demanding processes, neurons rely almost exclusively on the mitochondrial OXPHOS system to fulfill their energy needs^37,38^, which suggests that the OXPHOS system plays an important role in neural function. A recent study by Kimura et al.^39^ reported that low-frequency stimulation induces the accumulation of sarkosyl-insoluble tau aggregates in the hippocampus of aged WT mice, suggesting an important contribution of synaptic impairment to the formation of tau pathology. Taken together, mtDNA variants possibly cause disturbances in neural function, such as synaptic impairment, which may result in enhanced formation of tau pathology. Furthermore, mitochondrial oxidative stress caused by sod2 knockout was reported to induce hyperphosphorylation of tau in a Tg2576 mouse model of Alzheimer's disease^40^. Since the mitochondrial OXPHOS system also generates reactive oxygen species, such as superoxide anion radicals, mitochondria have antioxidant defense systems to maintain redox homeostasis in the cell. However, according to the findings of previous studies, an imbalance in redox homeostasis would contribute to the accumulation of tau pathology. Based on these pieces of evidence, distinct mtDNA variants in rTg4510 mice are considered to potentially explain mechanisms underlying phenotypic differences, such as variations in levels of tau pathology.

It is difficult to explain the mechanisms underlying gene expression patterns in rTg4510 mice. Fgf14 levels were expected to be reduced in rTg4510 mice due to transgene integrations. However, the results showed that Fgf14 levels in rTg4510 mice were much higher than in WT mice, which is in agreement with previous findings^41^. Dysregulation of Fgf14 mRNA splice variants has been suggested to contribute to the increase in Fgf14 levels^41^; however, the detailed mechanism remains unknown. Considering the findings of Fgf14 expression in rTg4510 mice, it is probable that unknown mechanisms may induce the expression of other genes, such as Vipr2, Wdr60, Esyt2, D430020J02Rik, and Ncapg2, although the expression levels of these genes are expected to be decreased due to transgene integrations. In addition, the result showing that Hspa1b expression in rTg4510_FxC mice was significantly lower than in rTg4510_CxF mice suggests that the downregulation of Hspa1b may contribute to severe pathological accumulation of tau in rTg4510_FxC mice, because Hspa1b (HSP70) has been reported to inhibit and degrade tau aggregation^42,43^.

The results of the present study raise concerns about the use of rTg4510 mice as models for the evaluation of therapeutic interventions of tau pathology. No study, except for our previous work^19^, has investigated tau pathology levels in reciprocal F1 hybrids of rTg4510 mice. We used Camk2α-tTA mice on a C57BL/6 J background, rather than the original 129S6 background^11,12^. Accordingly, differences in tau pathology levels may only occur in Camk2α-tTA mice on a C57BL/6 J background. In contrast, sex had no effect on pathological tau accumulation in the present study, while there was a sex difference in pathological tau accumulation in the original rTg4510 mice, which was more severe in females than males^20^. Furthermore, it has been confirmed that there are differences in the effect of the Y chromosome, mtDNA, maternal care, and environmental factors between reciprocal hybrid offspring. Therefore, it is important to take the genetic background into consideration when evaluating tau pathology in hybrid rTg4510 mice.

The results of this study revealed a strong effect of the genetic background on the differential accumulation of tau pathology between reciprocal F1 hybrids of rTg4510 mice, while there was no evident significant effect of sex. Comparisons between the two hybrids indicate that Mid1 may be associated with the differential accumulation of tau pathology. Hence, elucidation of the mechanisms underlying differences in tau pathology levels would provide novel insights into the accumulation of tau pathology in tauopathy. Furthermore, such findings would be beneficial to researchers developing therapeutic interventions for tauopathies.

## Methods

### Animals

All animal experiments complied with the ARRIVE guidelines, were carried out in accordance with the National Institutes of Health guide for the care and use of Laboratory animals (NIH Publications No. 8023, revised 1978), and were approved by the Animal Care and Use Committee at the Shiga University of Medical Science (approval no. 2018–6-5). All efforts were made to minimize animal suffering.

To obtain rTg4510 mice, the hemizygous Camk2α-tTA mouse line (on the C57BL/6 J background; 007,004; The Jackson Laboratory, Bar Harbor, ME, USA), carrying a tTA that consisted of the tet-off open reading frame placed downstream of Camk2α promoter elements, was crossbred with the hemizygous tetO-MAPT*P301L mouse line (on the FVB/NJ background; 015,815; The Jackson Laboratory) carrying human 0N4R tau cDNA with the P301L mutation placed downstream of a tetracycline-operon-responsive element. Consequently, mice harboring both the activator and responder genes were obtained and used as rTg4510 mice in the present study. The Camk2α-tTA mouse line on the C57BL/6 J background, rather than the original 129S6 background, was used in this study. Therefore, the mice obtained in this study had either the (C57BL/6 J × FVB/NJ)F1 or (FVB/NJ × C57BL/6 J)F1 background (Table 1). Littermates expressing neither the activator nor the responder were used as WTs. Two to four mice were housed per standard laboratory cage on wood shavings and fed a standard chow diet and maintained at 23ºC under a 12-h light/dark cycle (lights on at 08:00–20:00 h) with free access to water and food in a specific pathogen-free animal facility.

### Preparation of brain extracts

Mice were sacrificed at 3 and 6 months of age with an overdose injection of sodium pentobarbital (200 mg/kg, i.p.). Brains were quickly removed and cut into two hemispheres. Cerebral cortex tissues were isolated from the right hemisphere, snap frozen in liquid nitrogen, and stored at − 80 °C. Sarkosyl-insoluble tau was purified as described previously^44^ with slight modifications. Briefly, brain tissues were homogenized in 10 volumes of Tris-buffered saline [TBS; 25 mM Tris–HCl (pH 7.5), 150 mM NaCl, 1 mM EGTA, protease inhibitor cocktail (Roche Diagnostics, Mannheim, Germany) and phosphatase inhibitor cocktail (Roche Diagnostics)] and centrifuged in a TLA110 rotor (Beckman Coulter, Brea, CA, USA) at 27,433 × *g* for 20 min at 4 °C. The supernatant was collected as the TBS-soluble fraction. The pellet was resuspended in five volumes of high salt/sucrose buffer [10 mM Tris–HCl (pH 7.5), 0.8 M NaCl, 10% sucrose, and 1 mM EGTA]. The sample was then centrifuged at 27,433 × *g* for 20 min at 4 °C. The supernatant was transferred to a new tube, incubated in 1% sarkosyl at 37˚C for 1 h, and centrifuged at 149,361 × *g* for 1 h at 4 °C. The pellet was resuspended in 50 µL of TBS, sonicated, and collected as the sarkosyl-insoluble fraction.

### Western blot analysis

Western blot analysis was conducted as described previously^19^. The protein concentration of extracts was determined using a Protein Assay Bicinchoninate kit (Nacalai Tesque, Kyoto, Japan). TBS-soluble and sarkosyl-insoluble fractions were separated on precast polyacrylamide gels (Bolt Bis–Tris Plus Gels; Thermo Fisher Scientific, Waltham, MA, USA) and transferred to polyvinylidene difluoride membranes (iBlot 2 Transfer Stacks; Thermo Fisher Scientific). The membranes were blocked with 5% non-fat milk in TBS (25 mM Tris–HCl, pH 7.4, 0.9% NaCl) containing 0.1% Tween 20 for 1 h at room temperature and then incubated overnight at 4 °C with primary antibodies including mouse monoclonal antibodies against tau (clone HT7; 1:2000; Thermo Fisher Scientific); tau phosphorylated at Ser202 and Thr205 (clone AT8; 1:1000; Thermo Fisher Scientific), at Thr212 and Ser214 (clone AT100; 1:1000; Thermo Fisher Scientific), at Thr231 (clone AT180; 1:1000; Thermo Fisher Scientific), and at Thr181 (clone AT270; 1:1000; Thermo Fisher Scientific); this was followed by addition of β-actin (1:5000; Santa Cruz Biotechnology, Dallas, TX, USA), along with rabbit polyclonal anti-tau antibody (1:2,000,000; DAKO, Glostrup, Denmark), which was produced by immunization with recombinant human tau protein expressed in *Escherichia coli*, corresponding to the C-terminal part (amino acids 243–441) containing the four repeated sequences involved in microtubule binding. The membranes were further incubated with horseradish peroxidase-conjugated goat polyclonal antibody against mouse immunoglobulin (Ig)G (1:20,000; Jackson ImmunoResearch, West Grove, PA, USA) or rabbit IgG (1:20,000; Jackson ImmunoResearch) for 1 h. Immunoreactive proteins were visualized with a chemiluminescence substrate (SuperSignal West Pico PLUS Chemiluminescent Substrate; Thermo Fisher Scientific) using a lumino-image analyzer (LAS-4000 mini; Fujifilm, Tokyo, Japan). The band density was analyzed using image processing software (ImageJ; National Institutes of Health, Bethesda, MD, USA). The amount of sample load in the sarkosyl-insoluble fraction was based on the initial wet weight of the extracted brain tissues.

### Immunohistochemical analysis

The brain hemisphere was fixed in 4% paraformaldehyde for 24 h at 4 °C, immersed in 0.1 M phosphate buffer (pH 7.4) containing 15% sucrose and 0.1% sodium azide for at least 2 days for cryoprotection, and subsequently cut into 20-µm coronal sections with the use of a cryostat. Immunohistochemical analysis was performed as described in previous studies^19,45^. Briefly, free-floating sections were incubated with biotinylated antibodies against phosphorylated tau (clone AT8; 1:1000; Thermo Fisher Scientific) and tau (clone HT7; 1:2000; Thermo Fisher Scientific) in PBS-T containing 0.2% bovine serum albumin overnight at 4 °C. The antibodies were detected using a Vectastain ABC Elite kit (1:1000; Vector Laboratories, Burlingame, CA, USA) with 3,3′-diaminobenzidine (Dojindo Laboratories, Kumamoto, Japan) with nickel ammonium sulfate. The sections were scanned with a camera and the immunoreactive areas were quantified using ImageJ software.

### Simplified Gallyas silver staining

Simplified Gallyas staining was performed as described in a previous study^46^ with some modifications^19,45^.

### RNA isolation and quantitative polymerase chain reaction (qPCR)

Total RNA was extracted from the cerebral cortex using an RNeasy Plus Universal Mini Kit (Qiagen, Hilden, Germany) in accordance with the manufacturer’s instructions. RNA samples were reverse transcribed into cDNA using SuperScript IV VILO Master Mix (Thermo Fisher Scientific) in accordance with the manufacturer’s instructions. qPCR was conducted in duplicate 10-µL reactions containing 5 μl of THUNDERBIRD SYBR qPCR Mix (Toyobo, Osaka, Japan), 0.3 µM of each primer, and 0.5 ng of cDNA on a LightCycler 480 instrument (Roche Diagnostics) using the following cycling parameters: denaturation at 95 °C for 1 min, followed by 45 cycles of annealing at 95 °C for 15 s and extension at 60 °C for 30 s. Melting curve analysis confirmed that single amplicons were present and relative gene expression levels were determined using the ΔΔCT method with glyceraldehyde-3-phosphate dehydrogenase as the reference gene. Primer sequences are listed in Supplementary Table S5.

### Plasmid construction and stable cell line development

Full-length human tau (0N4R) was cloned into the pcDNA3.1 vector acquired from human neuroblastoma cell line SH-SY5Y cDNA. A site-directed mutation at P301L was introduced in the construct using the QuikChange Lightning Multi Site-Directed Mutagenesis Kit (Agilent Technologies, Santa Clara, CA, USA). The sequence of human tau with the P301L mutation was then subcloned into the pIRESpuro3 vector (Clontech Laboratories, Mountain View, CA, USA).

HEK293T cells were cultured in Dulbecco’s modified Eagle’s medium (Nacalai Tesque) supplemented with 10% (*v*/*v*) fetal bovine serum (Hyclone, Logan UT, USA), and kept at 37 °C under a humidified atmosphere of 5% CO_2_/95% air. Cells were seeded at 1 × 10^5^ cells/well in a 24-well plate. At 24 h after plating, the cells were transfected with the pIRESpuro3 vector containing human tau with the P301L mutation using Lipofectamine 3000 reagent (Thermo Fisher Scientific). After a 24-h incubation period, the cells were transferred to a 6-cm culture dish, and stable cell lines were selected with 1 µg/mL puromycin (Nacalai Tesque) for 12 days. Single cells from resistant colonies were transferred into the wells of 96-well plates.

### *MID1* silencing with siRNA

HEK293T cells were cultured in Dulbecco’s modified Eagle’s medium (Nacalai Tesque) supplemented with 10% (*v*/*v*) fetal bovine serum (Hyclone, Logan UT, USA), and kept at 37 °C under a humidified atmosphere of 5% CO_2_/95% air. Cells were seeded at 1 × 10^5^ cells/well in a 24-well plate. At 24 h after plating, the cells were transfected with the pIRESpuro3 vector containing human tau with the P301L mutation using Lipofectamine 3000 reagent (Thermo Fisher Scientific). After a 24-h incubation period, the cells were transferred to a 6-cm culture dish, and stable cell lines were selected with 1 µg/mL puromycin (Nacalai Tesque) for 12 days. Single cells from resistant colonies were transferred into the wells of 96-well plates.

### Statistical analysis

Statistical analyses were performed using GraphPad Prism 7 software (GraphPad Software, La Jolla, CA, USA). Data are presented as the mean ± standard error of the mean (SEM). Two-way ANOVA with genetic background and sex as factors was conducted. If the results of the two-way ANOVA revealed a main effect of the genetic background or sex, statistical significance was subsequently determined with the Mann–Whitney *U* test for the corresponding single comparison. The Student’s *t*-test was used to identify significant differences in the results of the cell culture experiments. A probability (*p*) value of < 0.05 was considered statistically significant.

## Supplementary Information

Supplementary Information
