# Supplementary material for: Differential accumulation of tau pathology between reciprocal F1 hybrids of rTg4510 mice
Source: Sci Rep. 2021 May 5;11:9623. doi: 10.1038/s41598-021-89142-2 (PMC8100160; doi:10.1038/s41598-021-89142-2)
Supplement: Supplementary file 1 — Supplementary Information [file 41598_2021_89142_MOESM1_ESM.pdf]

Supplementary Information

Differential accumulation of tau pathology between reciprocal F1 hybrids of rTg4510 mice

Daijiro Yanagisawa, Hamizah Shahirah Hamezah, Aslina Pahrudin Arrozi, Ikuo Tooyama

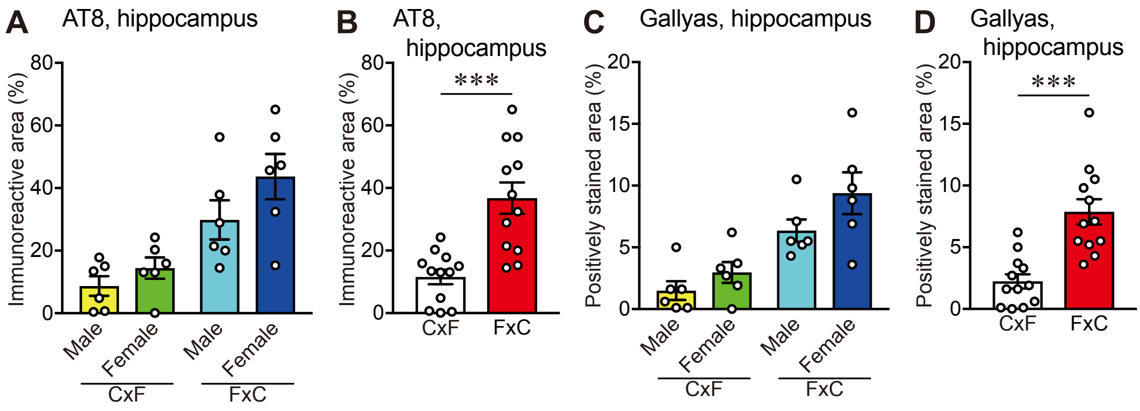

Supplementary Figure S1. Quantitative analysis of the AT8-immunoreactive area and Gallyas-positively-stained area in the hippocampus at 6 months of age. Two-way ANOVA (genetic background × sex) revealed a main effect of background but no effect of sex on the AT8-immunoreactive area (A) and Gallyas-positively stained area (C) (Supplementary Table S1). A Mann-Whitney test revealed significantly higher levels of AT8-immunoreactive area (B) and Gallyas-positively stained area (D) in rTg4510_FxCmice than rTg4510_CxF mice. The rTg4510_CxF and rTg4510_FxC groups contained six males and six females each (total n = 12 in each group). Data are presented as mean ± SEM. Significance (Mann-Whitney test): ***$P < 0.001$.

Supplementary Figure S2

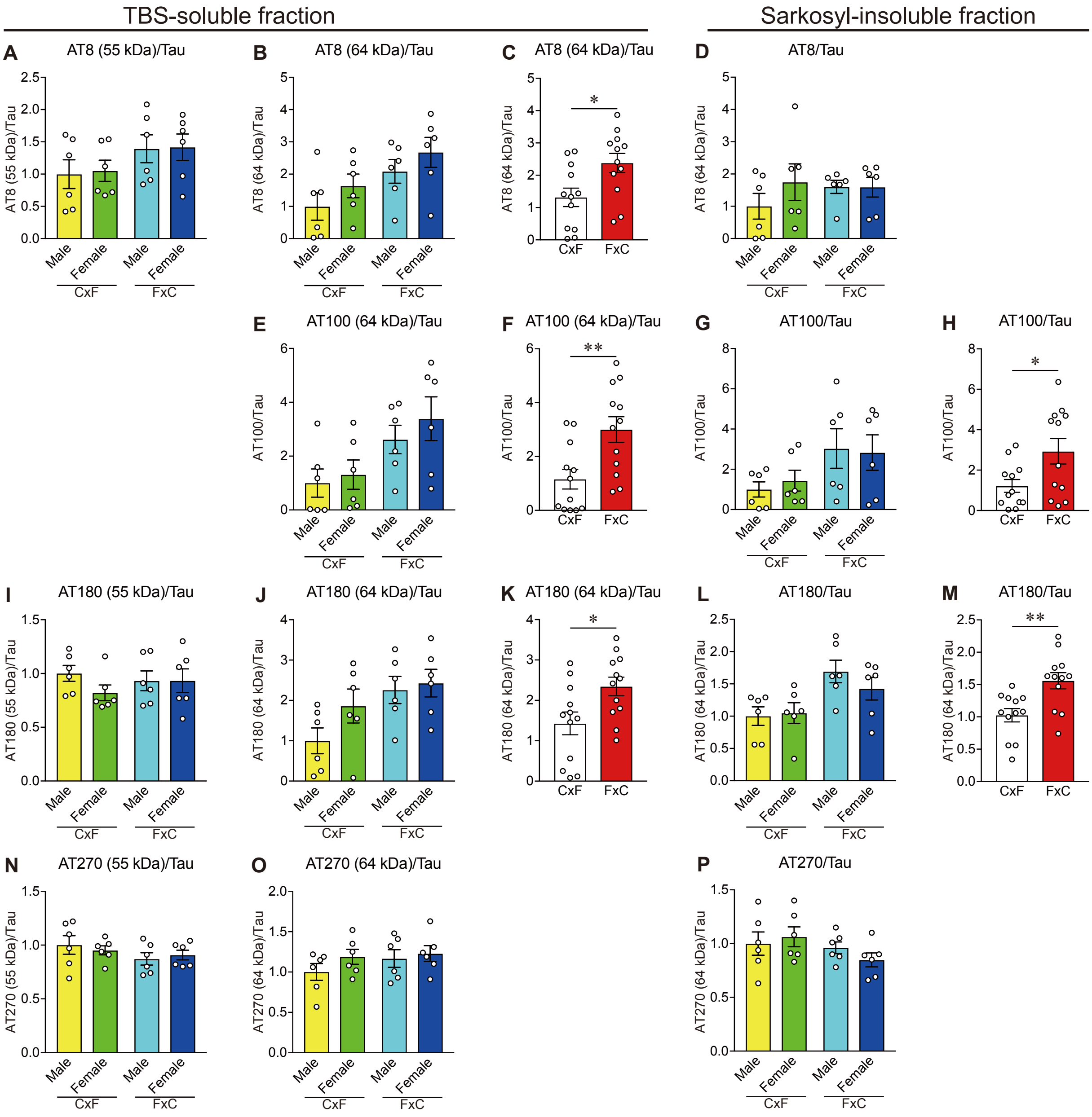

Supplementary Figure S2. The ratios of tau phosphorylation. The ratios of phosphorylated tau at the AT8 (A–D), AT100 (E–H), AT280 (I–M), and AT270 (N–P) sites to total tau were determined at 55 kDa (A, I, N) and 64 kDa (B, C, E, F, J, K, O) in the TBS-soluble fraction and at 64 kDa (D, G, H, L, M, P) in the sarkosyl-insoluble fraction of rTg4510 mice at 6 months of age. The rTg4510_CxF and rTg4510_FxC groups contained six males and six

females each (n = 12/group). Data are presented as the mean ± SEM. $^{**}p < 0.01$, $^{*}p < 0.05$ (Mann–Whitney U test).

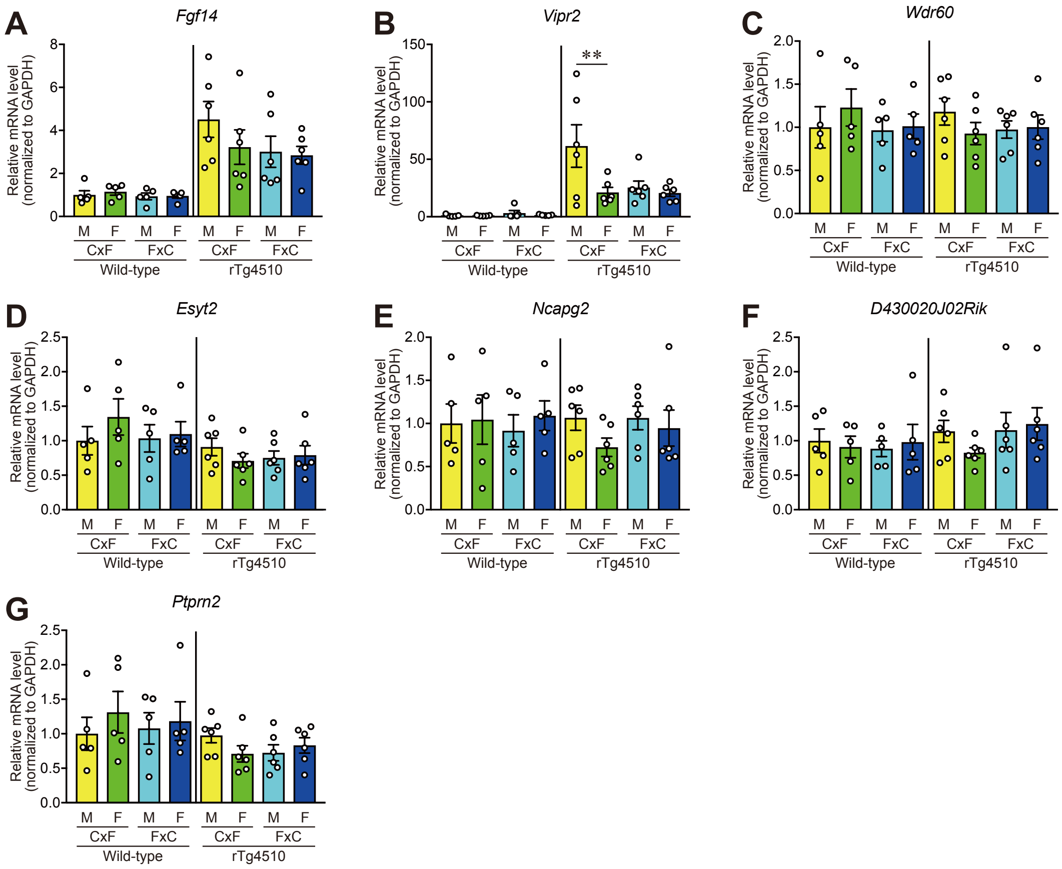

Supplementary Figure S3. Expression levels of genes affected by the insertion of transgenes to rTg4510 mice at 6 month of age. qPCR analyses of *Fgf14* (A), *Vipr2* (B), *Wrd60* (C), *Esyt2* (D), *Ncapg2* (E), *D430020J02Rik* (F), and *Ptprn2* (G) were conducted in wild-type and rTg4510 mice. Two-way ANOVA (genetic background × sex) revealed no effects of background or sex on the levels of mRNA expression for these genes (Supplementary Table S2 and S3). The wild-type_CxF and wild-type_FxC groups contained five males and five females each (n = 10 in each group). The rTg4510_CxF and rTg4510_FxC groups contained six males and six females each (n = 12 in each group). Data are presented as mean ± SEM.

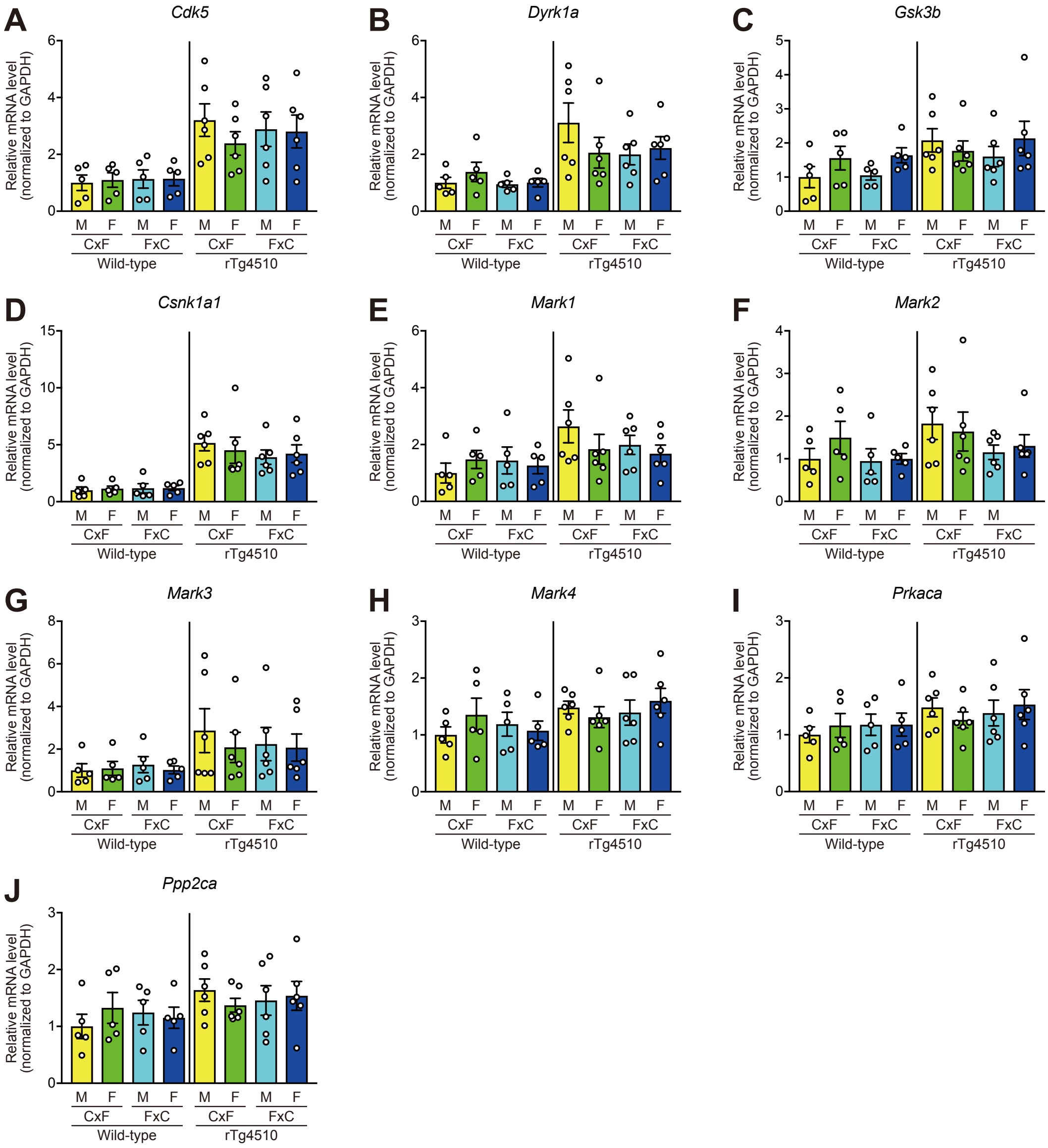

Supplementary Figure S4. Expression levels of kinases and a phosphatase related to the formation of phosphorylated tau at 6 month of age. qPCR analyses of *Cdk5* (A), *Dyrk1a* (B), *Gskb* (C), *Csnk1a1* (D), *Mark1* (E), *Mark2* (F), *Mark3* (G), *Mark4* (H), *Prkaca* (I), and *Ppp2ca* (J) were conducted in wild-type and rTg4510 mice. Two-way ANOVA (genetic background × sex) revealed no effects of background or sex on the levels of mRNA expression for these genes (Supplementary Table S2 and S3). The wild-type_CxF and wild-

type_FxC groups contained five males and five females each (n = 10 in each group). The rTg4510_CxF and rTg4510_FxC groups contained six males and six females each (n = 12 in each group). Data are presented as mean ± SEM.

Supplementary Figure S5

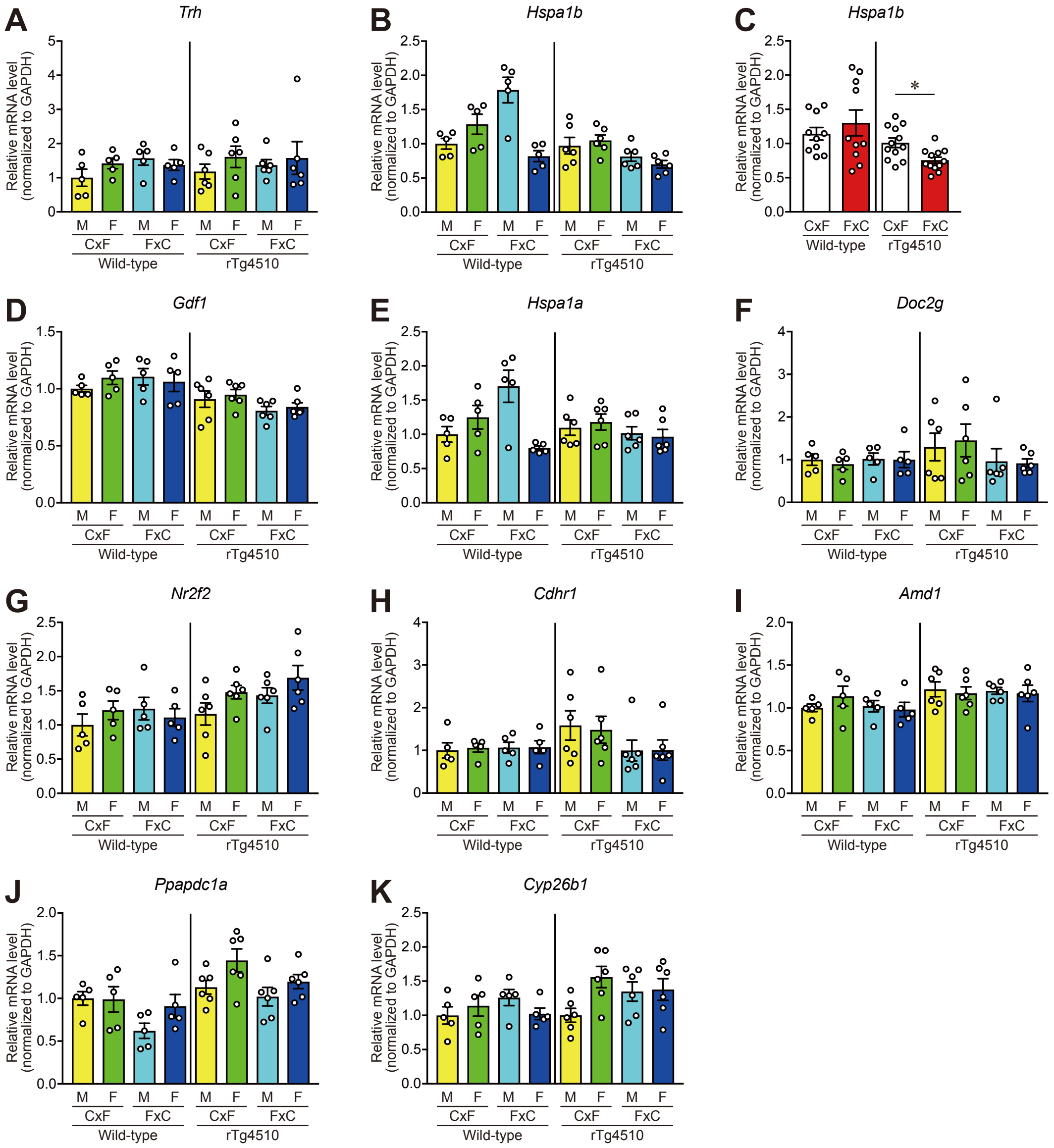

Supplementary Figure S5. Expression levels of the top 10 upregulated genes selected according to the RNA-seq result between wild-type_CxF and wild-type_FxC at 6 month of age. qPCR analyses of *Trh* (A), *Hspa1b* (B, C), *Gdf1* (D), *Hspa1a* (E), *Doc2g* (F), *Nr2f2* (G), *Cdhr1* (H), *Amd1* (I), *Ppapdc1a* (J), and *Cyp26b1* (K) were conducted in wild-type and rTg4510 mice. (B) Two-way ANOVA (genetic background × sex) of qPCR analysis results revealed a main effect of background but no effect of sex on *Hspa1b* mRNA levels (B) in

rTg4510 mice (Supplementary Table S2). (C) A Mann-Whitney test revealed a significant difference in *Hspa1b* mRNA levels between rTg4510_CxF and rTg4510_FxC mice. The wild-type_CxF and wild-type_FxC groups contained five males and five females each (n = 10 in each group). The rTg4510_CxF and rTg4510_FxC groups contained six males and six females each (n = 12 in each group). Data are presented as mean ± SEM. Significance (Mann-Whitney test): *P < 0.05.

Supplementary Figure S6

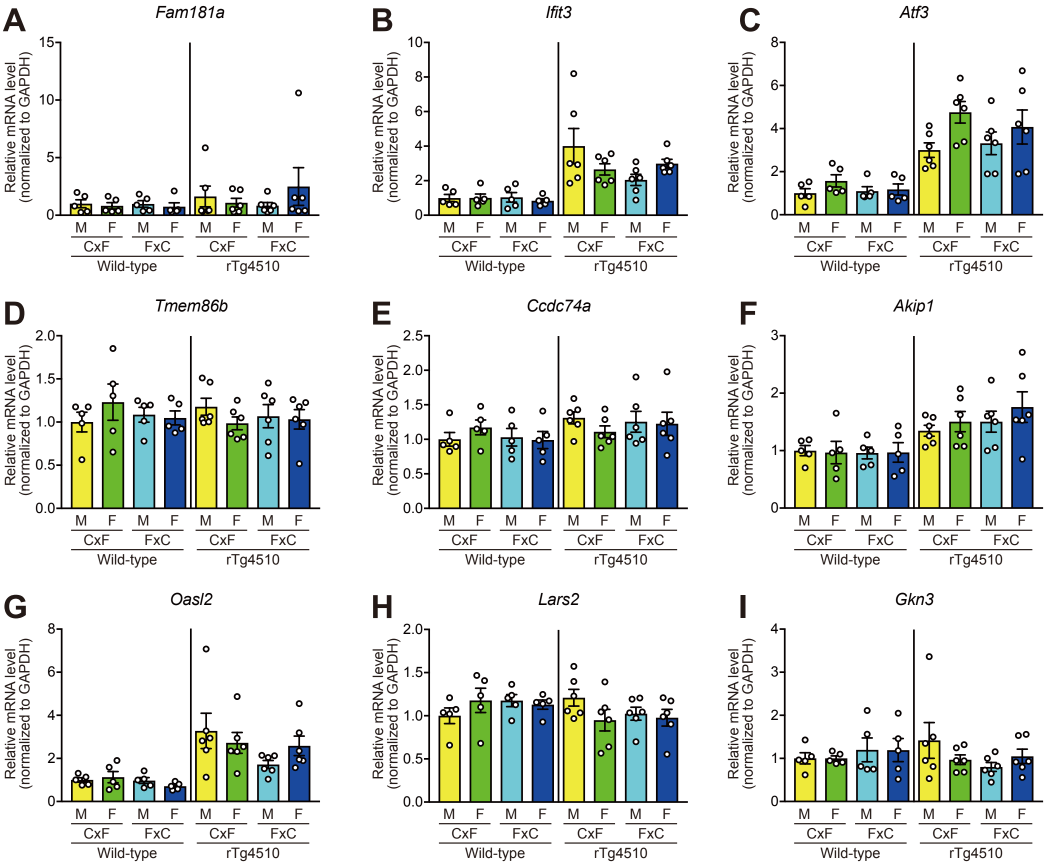

Supplementary Figure S6. Expression levels of the top 10 downregulated genes selected according to the RNA-seq result between wild-type_CxF and wild-type_FxC at 6 month of age. qPCR analyses of *Fam181a* (A), *Ifit3* (B), *Atf3* (C), *Trem86b* (D), *Ccdc74a* (E), *Akip1* (F), *Oasl2* (G), *Lars2* (H), and *Gkn3* (I) were conducted in wild-type and rTg4510 mice. Two-way ANOVA (genetic background × sex) revealed no effects of background or sex on the levels of mRNA expression for these genes (Supplementary Table S2 and S3). The wild-type_CxF and wild-type_FxC groups contained five males and five females each (n = 10 in each group). The rTg4510_CxF and rTg4510_FxC groups contained six males and six females each (n = 12 in each group). Data are presented as mean ± SEM.

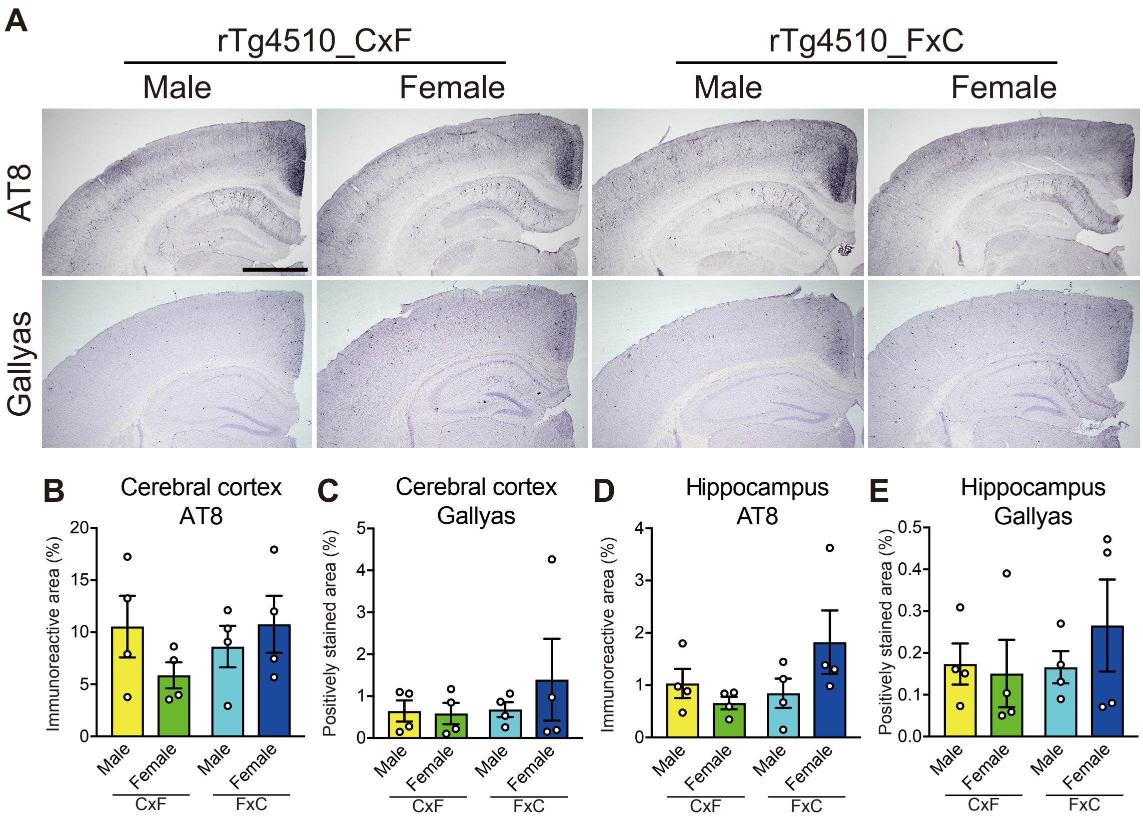

Supplementary Figure S7. Histological analysis of rTg4510 mice at 3 months of age. (A) Representative photographs showing immunohistochemistry for phosphorylated tau (clone AT8) and Gallyas silver staining. Scale bar: 1 mm. (B–E) Quantitative analysis showed that there were no significant differences in AT8-immunoreactive area (B, D) and Gallyas-positively stained area (C, E) in the cerebral cortex (B, C) and hippocampus (D, E).

Supplementary Figure S8

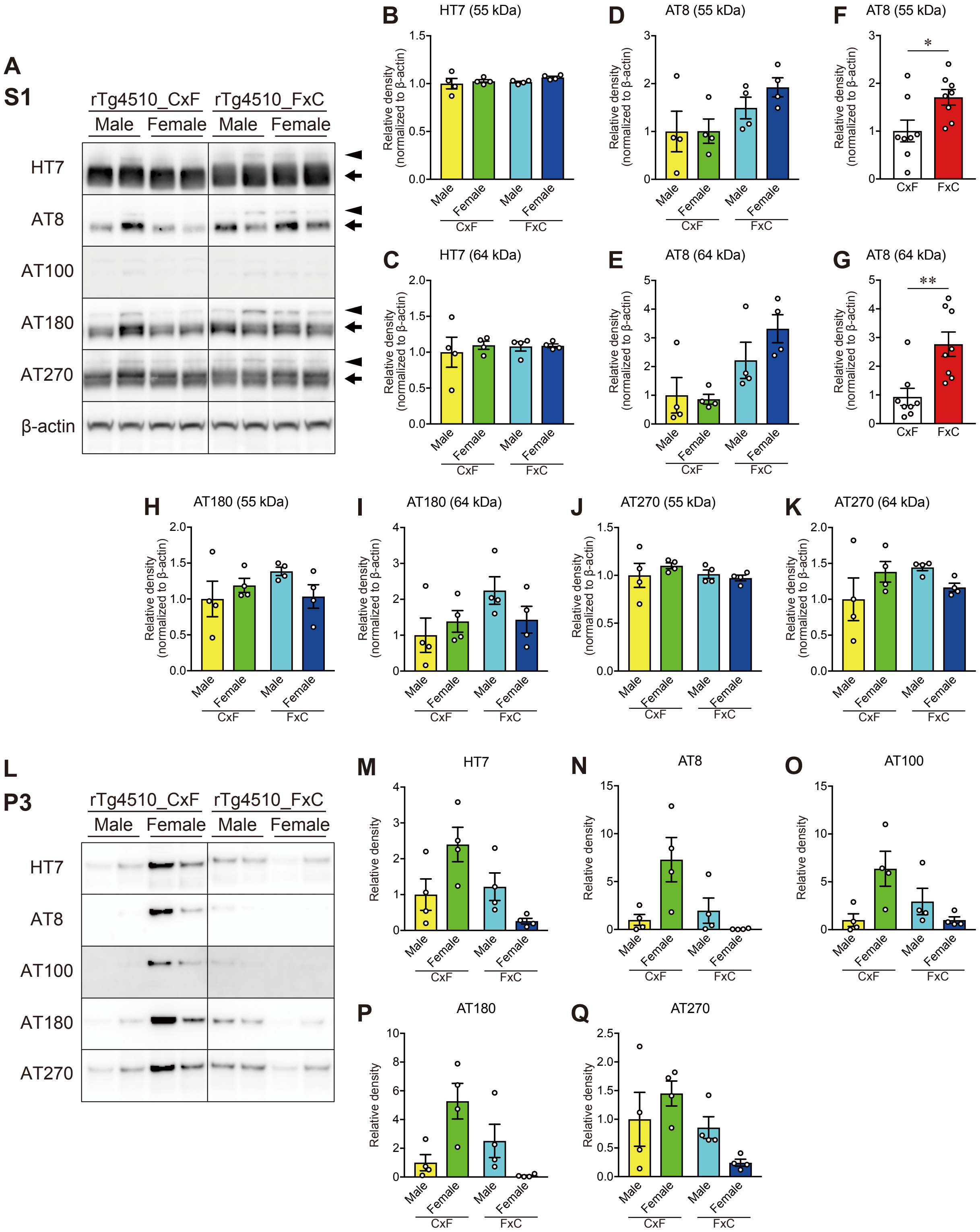

Supplementary Figure S8. The levels of soluble and insoluble tau in rTg4510 mice at 3 months of age. (A) Representative images of western blots for HT7, AT8, AT100 (not detectable), AT180, AT270, and β-actin in the TBS-soluble fraction of the cerebral cortex. The band images in each row were obtained from the same gel but CxF and FxC were separated. Arrows and arrowheads indicate bands at 55 kDa and 64 kDa, respectively. Full-length blots are presented in Supplementary Figure S12. (B–K) Densitometric analysis of tau accumulation in the TBS-soluble fraction. Two-way ANOVA (genetic background × sex) revealed no effects of genetic background or sex on the levels of HT7, AT180, and AT270, except for AT8. In two-way ANOVA, there was a main effect of genetic background but no effect of sex on the levels of AT8 at 55 kDa (D) and 64 kDa (E). A Mann-Whitney test revealed significantly high levels of AT8 at 55 kDa (F) and 64 kDa (G) in rTg4510_FxC mice, compared with rTg4510_CxF mice. (L) Representative images of western blots for HT7, AT8, AT180, and AT270 in the sarkosyl-insoluble fraction of the cerebral cortex. The band images in each row were obtained from the same gel but CxF and FxC were separated. Full-length blots are presented in Supplementary Figure S12. (M–Q) Densitometric analysis of tau accumulation in the sarkosyl-insoluble fraction. Two-way ANOVA (genetic background × sex) revealed no effects of genetic background or sex on the levels of HT7, AT8, AT100, AT180, AT270 between rTg4510_CxF mice and rTg4510_FxC mice. The rTg4510_CxF and rTg4510_FxC groups contained four males and four females each (total n = 8 in each group). Data are presented as mean ± SEM. Significance (Mann-Whitney test): $^{**}P < 0.01$, $^{*}P < 0.05$.

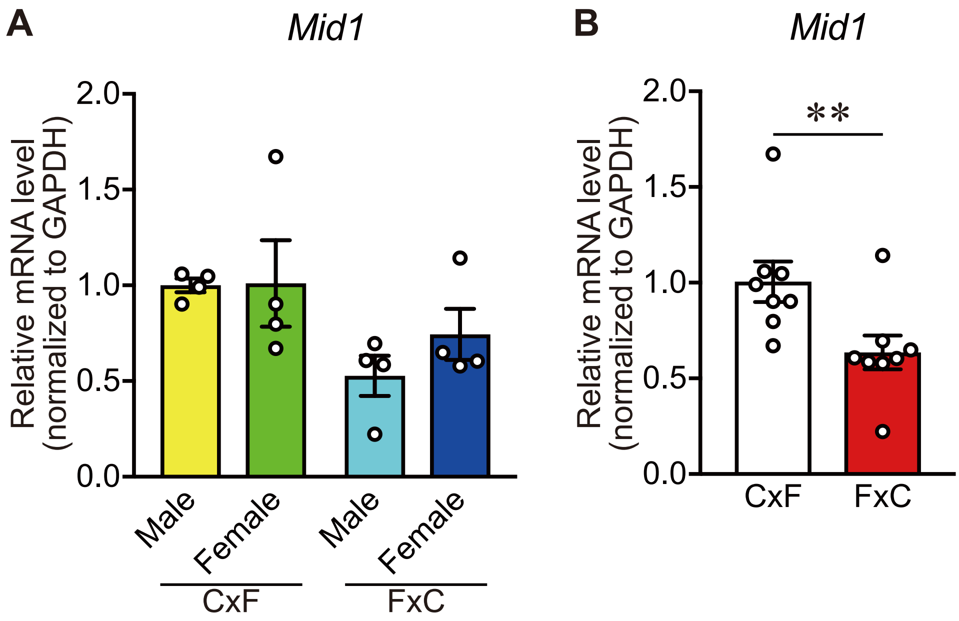

Supplementary Figure S9. The level of *midline-1* mRNA in rTg4510 mice at 3 months of age. (A) Two-way ANOVA (genetic background × sex) of the qPCR results revealed a main effect of genetic background but no effect of sex on the mRNA levels of *midline-1* in rTg4510 mice. (B) Mann-Whitney test revealed significantly lower *midline-1* mRNA levels in rTg4510_FxC, compared with rTg4510_CxF mice. The rTg4510_CxF and rTg4510_FxC groups contained four males and four females each (n = 8 in each group). Data are presented as mean ± SEM. Significance (Mann-Whitney test): **P < 0.01.

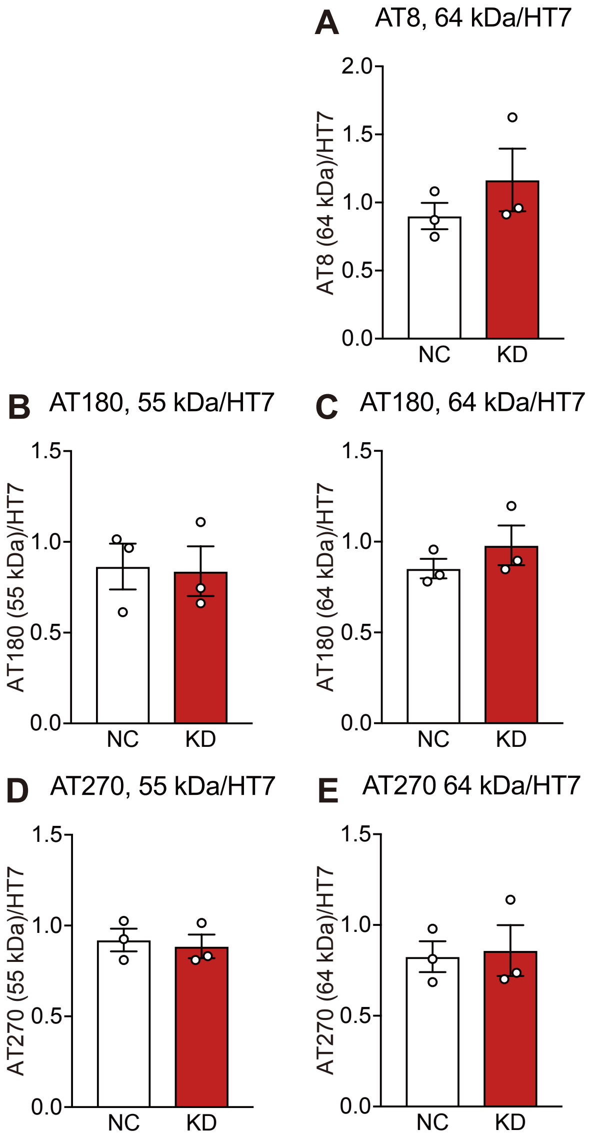

Supplementary Figure S10. The ratios of phosphorylated tau to total tau between negative control siRNA and MID1 siRNA treatment in HEK293T cells stably expressing human tau containing a P301L mutation. There were no significant differences in the ratios of AT8, AT180, and AT270 to HT7 between cells treated with negative control siRNA and MID1 siRNA.

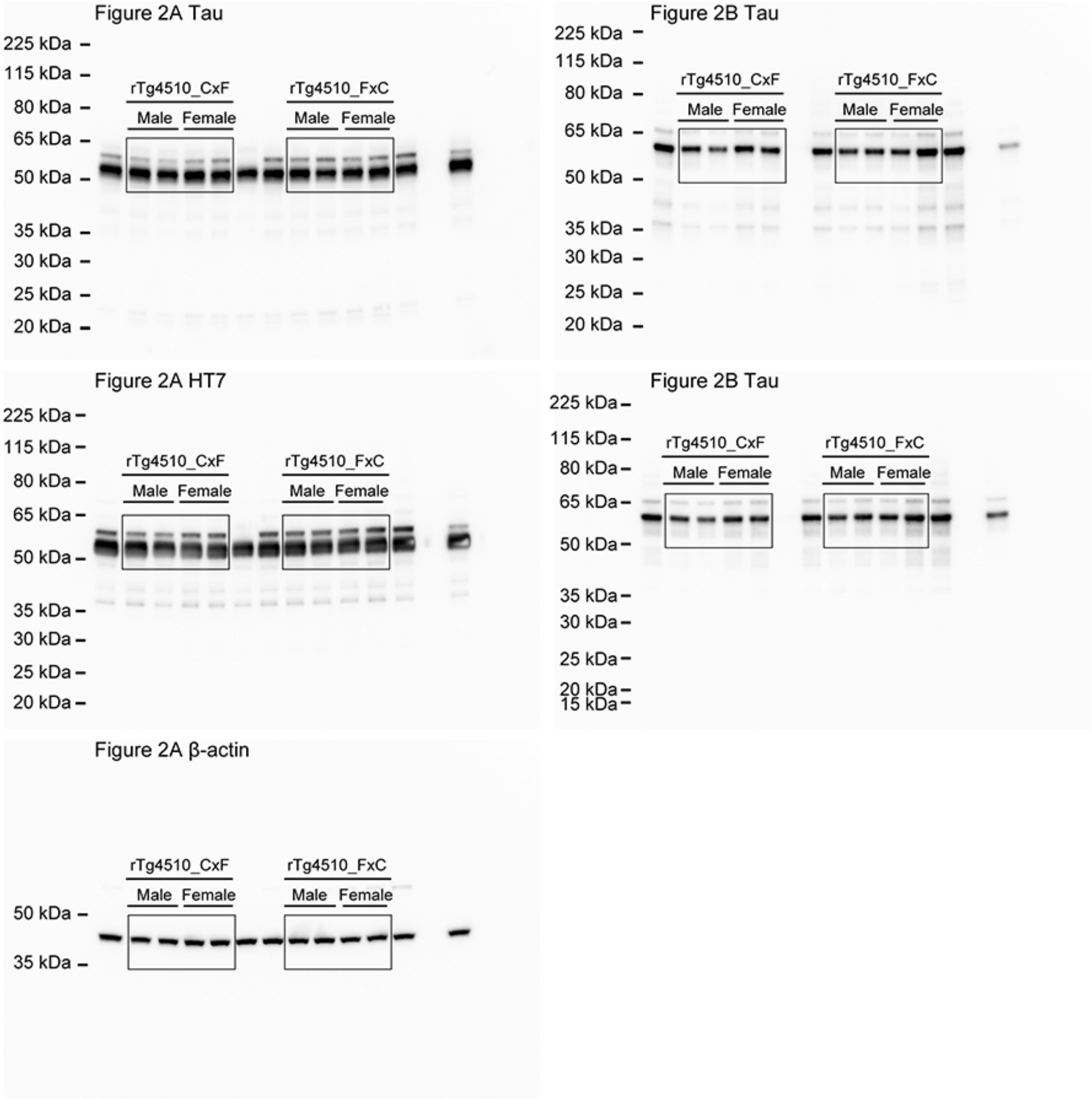

Supplementary Figure S11. Full-length blots in Figure 2.

Supplementary Figure S12

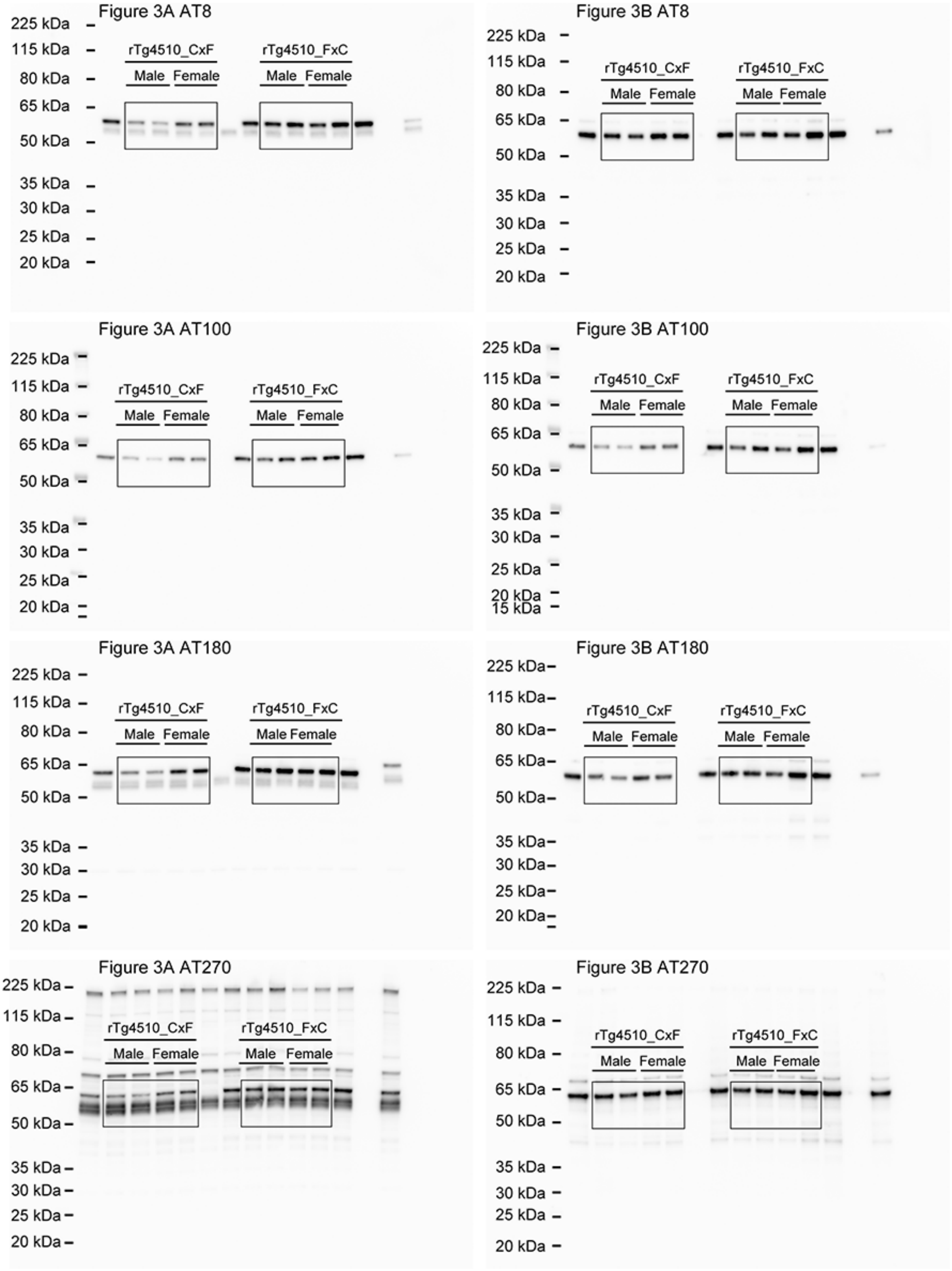

Supplementary Figure S12. Full-length blots in Figure 3.

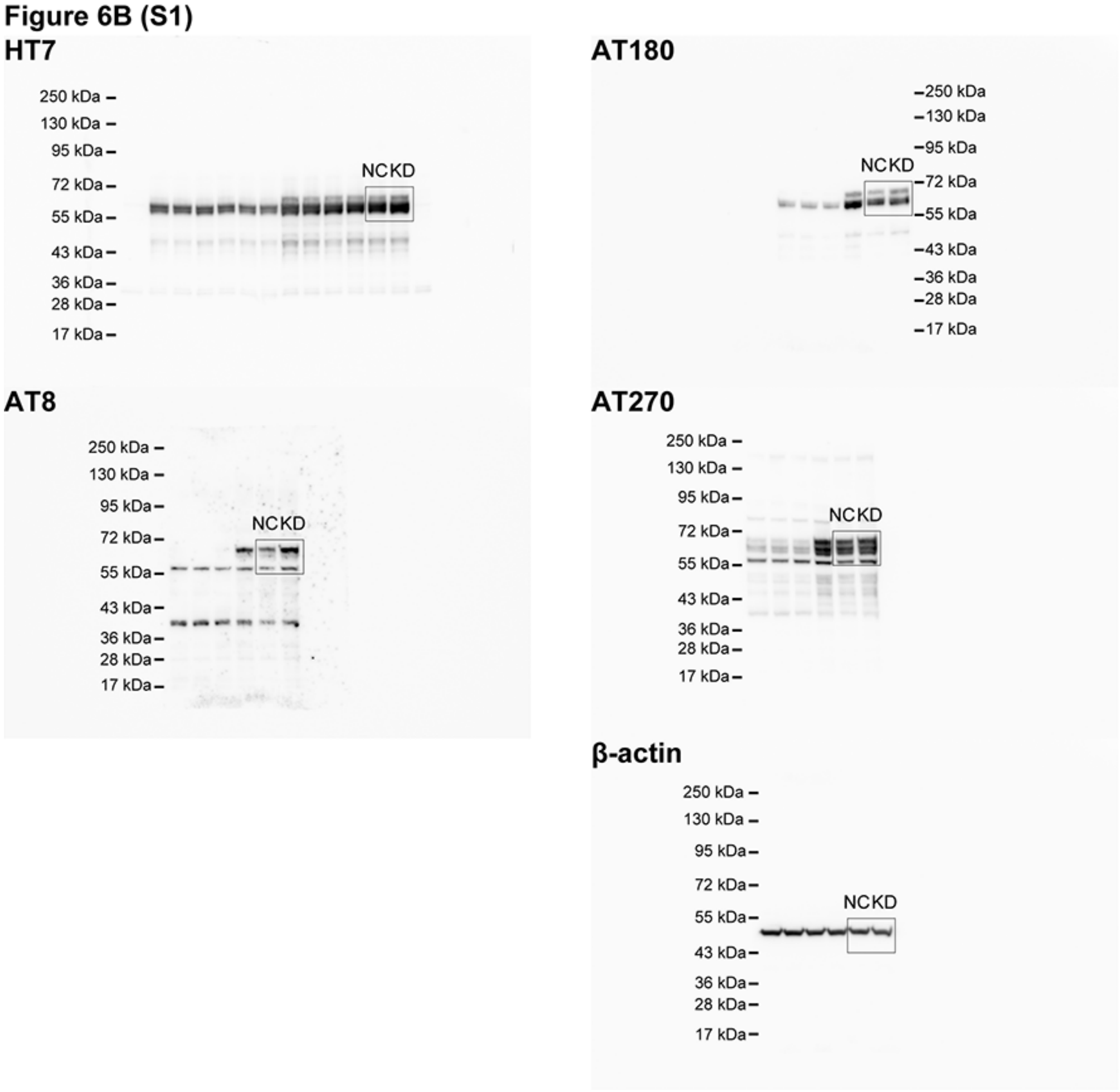

Supplementary Figure S13. Full-length blots in Figure 6B.

Supplementary Figure S14

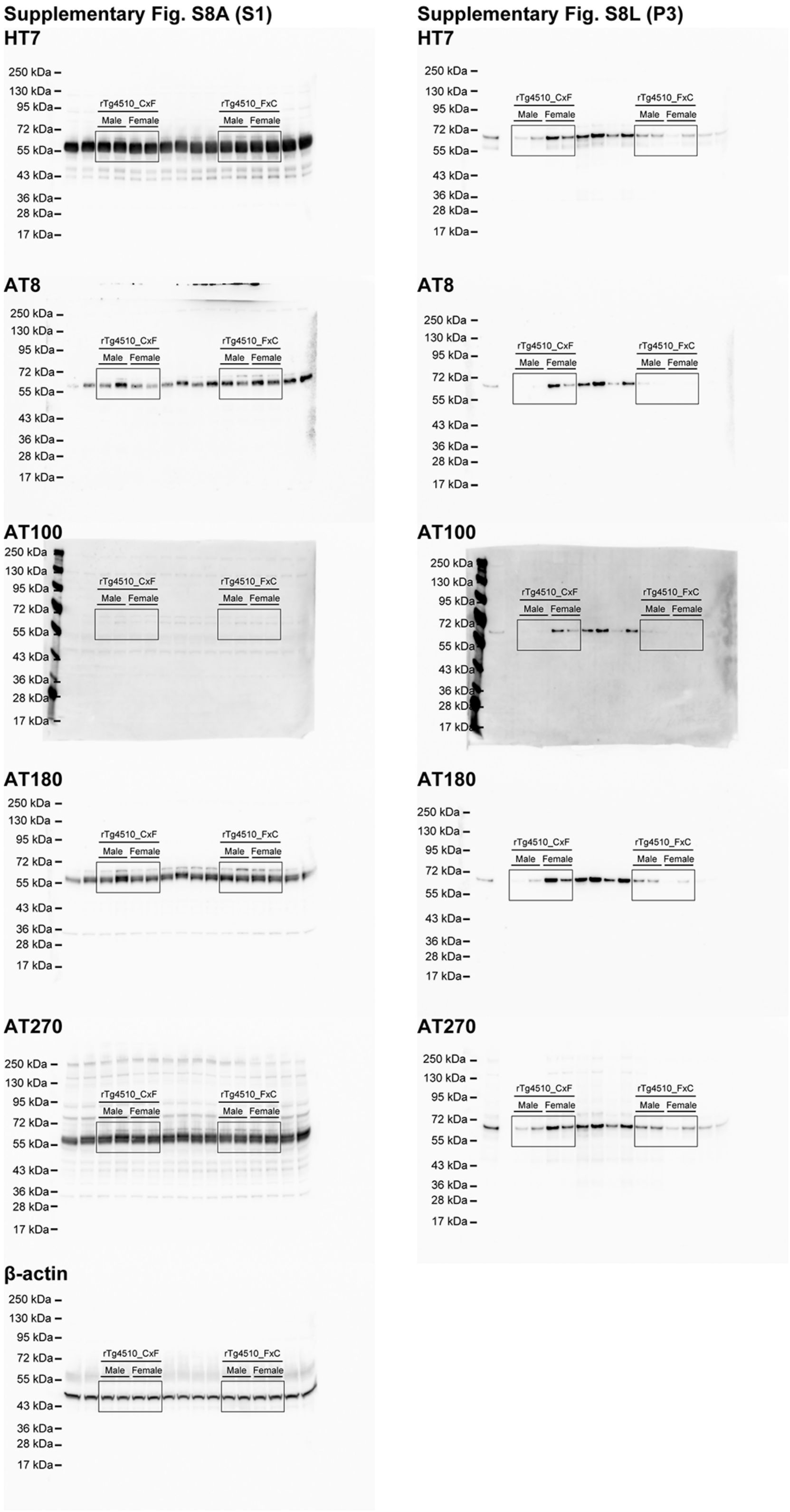

Supplementary Figure S14. Full-length blots in Supplementary Figure S8.

Supplementary Table S1. Two-way ANOVA of background and sex effects on the progression of tau pathology in rTg4510 mice.

		Background		Sex		Interaction	
		F (DFn, DFd)	P value	F (DFn, DFd)	P value	F (DFn, DFd)	P value
Histological analysis	AT8 in cc	F (1, 20) = 31.18	P<0.0001	F (1, 20) = 3.37	P=0.0813	F (1, 20) = 0.04933	P=0.8265
	AT8 in hp	F (1, 20) = 22.6	P=0.0001	F (1, 20) = 3.419	P=0.0793	F (1, 20) = 0.5839	P=0.4537
	Gal in cc	F (1, 20) = 45.74	P<0.0001	F (1, 20) = 3.726	P=0.0679	F (1, 20) = 0.3895	P=0.5396
	Gal in hp	F (1, 20) = 25.53	P<0.0001	F (1, 20) = 4.073	P=0.0572	F (1, 20) = 0.4937	P=0.4904
Western blotting (S1)	Tau (55 kDa)	F (1, 20) = 0.495	P=0.4898	F (1, 20) = 0.2426	P=0.6277	F (1, 20) = 3.476	P=0.0770
	Tau (64 kDa)	F (1, 20) = 17.12	P=0.0005	F (1, 20) = 1.118	P=0.3030	F (1, 20) = 1.385	P=0.2531
	HT7 (55 kDa)	F (1, 20) = 0.5535	P=0.4656	F (1, 20) = 0.3885	P=0.5401	F (1, 20) = 2.587	P=0.1234
	HT7 (64 kDa)	F (1, 20) = 23.49	P<0.0001	F (1, 20) = 3.783	P=0.0660	F (1, 20) = 0.04276	P=0.8383
	AT8 (55 kDa)	F (1, 20) = 2.538	P=0.1268	F (1, 20) = 0.01865	P=0.8927	F (1, 20) = 0.7056	P=0.4108
	AT8 (64 kDa)	F (1, 20) = 7.854	P=0.0110	F (1, 20) = 0.8822	P=0.3588	F (1, 20) = 0.09279	P=0.7638
	AT100 (64 kDa)	F (1, 20) = 10.81	P=0.0037	F (1, 20) = 1.425	P=0.2466	F (1, 20) = 0.5826	P=0.4542
	AT180 (55 kDa)	F (1, 20) = 1.757	P=0.2000	F (1, 20) = 0.3825	P=0.5432	F (1, 20) = 3.516	P=0.0755
	AT180 (64 kDa)	F (1, 20) = 12.75	P=0.0019	F (1, 20) = 2.527	P=0.1276	F (1, 20) = 0.03419	P=0.8552
	AT270 (55 kDa)	F (1, 20) = 0.2224	P=0.6424	F (1, 20) = 0.197	P=0.6619	F (1, 20) = 0.2224	P=0.6424
	AT270 (64 kDa)	F (1, 20) = 19.72	P=0.0003	F (1, 20) = 3.219	P=0.0879	F (1, 20) = 0.1689	P=0.6855
Western blotting (P3)	Tau	F (1, 20) = 2.553	P=0.1258	F (1, 20) = 0.3384	P=0.5672	F (1, 20) = 1.223	P=0.2818
	HT7	F (1, 20) = 5.937	P=0.0243	F (1, 20) = 0.7476	P=0.3975	F (1, 20) = 0.5607	P=0.4627
	AT8	F (1, 20) = 2.003	P=0.1723	F (1, 20) = 0.4126	P=0.5279	F (1, 20) = 0.6812	P=0.4189
	AT100	F (1, 20) = 4.72	P=0.0420	F (1, 20) = 0.3025	P=0.5884	F (1, 20) = 0.1345	P=0.7177
	AT180	F (1, 20) = 4.868	P=0.0392	F (1, 20) = 0.1474	P=0.7051	F (1, 20) = 0.3782	P=0.5455
	AT270	F (1, 20) = 2.876	P=0.1054	F (1, 20) = 0.3694	P=0.5501	F (1, 20) = 0.4376	P=0.5158

Supplementary Table S2. Two-way ANOVA of background and sex effects on gene expression in rTg4510 mice.

	Background		Sex		Interaction	
	F (DFn, DFd)	P value	F (DFn, DFd)	P value	F (DFn, DFd)	P value
P301L tau	F (1, 20) = 1.086	P=0.3099	F (1, 20) = 1.916	P=0.1816	F (1, 20) = 0.3025	P=0.5884
Mouse tau	F (1, 20) = 1.765	P=0.1990	F (1, 20) = 2.929e-005	P=0.9957	F (1, 20) = 0.2929	P=0.5944
Gapdh	F (1, 20) = 0.2322	P=0.6351	F (1, 20) = 3.397	P=0.0802	F (1, 20) = 0.7986	P=0.3822
Fgf14	F (1, 20) = 1.756	P=0.2000	F (1, 20) = 1.044	P=0.3192	F (1, 20) = 0.6236	P=0.4390
Vipr2	F (1, 20) = 3.313	P=0.0837	F (1, 20) = 5.071	P=0.0357	F (1, 20) = 3.168	P=0.0903
Wdr60	F (1, 20) = 0.2451	P=0.6259	F (1, 20) = 0.7196	P=0.4063	F (1, 20) = 1.129	P=0.3006
Esyt2	F (1, 20) = 0.09778	P=0.7577	F (1, 20) = 0.4787	P=0.4970	F (1, 20) = 1.047	P=0.3185
Ncapg2	F (1, 20) = 0.5142	P=0.4816	F (1, 20) = 2.236	P=0.1504	F (1, 20) = 0.5283	P=0.4757
D430020J02Rik	F (1, 20) = 1.248	P=0.2773	F (1, 20) = 0.3232	P=0.5760	F (1, 20) = 1.057	P=0.3162
Ptprn2	F (1, 20) = 0.3234	P=0.5759	F (1, 20) = 0.4898	P=0.4921	F (1, 20) = 2.737	P=0.1136
Cdk5	F (1, 20) = 0.008325	P=0.9282	F (1, 20) = 0.6753	P=0.4209	F (1, 20) = 0.4559	P=0.5073
Dyrk1a	F (1, 20) = 0.843	P=0.3695	F (1, 20) = 0.6396	P=0.4333	F (1, 20) = 1.541	P=0.2288
Gsk3b	F (1, 20) = 0.02073	P=0.8870	F (1, 20) = 0.09231	P=0.7644	F (1, 20) = 1.304	P=0.2670
Csnk1a1	F (1, 20) = 0.8573	P=0.3655	F (1, 20) = 0.03977	P=0.8440	F (1, 20) = 0.3205	P=0.5776
Mark1	F (1, 20) = 0.8005	P=0.3816	F (1, 20) = 1.518	P=0.2323	F (1, 20) = 0.2981	P=0.5911
Mark2	F (1, 20) = 2.282	P=0.1465	F (1, 20) = 0.003499	P=0.9534	F (1, 20) = 0.2515	P=0.6215
Mark3	F (1, 20) = 0.1601	P=0.6933	F (1, 20) = 0.3504	P=0.5605	F (1, 20) = 0.1509	P=0.7018
Mark4	F (1, 20) = 0.2749	P=0.6059	F (1, 20) = 0.009293	P=0.9242	F (1, 20) = 0.9906	P=0.3315
Prkaca	F (1, 20) = 0.1723	P=0.6825	F (1, 20) = 0.03005	P=0.8641	F (1, 20) = 0.8116	P=0.3784
Ppp2ca	F (1, 20) = 0.001135	P=0.9735	F (1, 20) = 0.1878	P=0.6694	F (1, 20) = 0.6534	P=0.4284
Trh	F (1, 20) = 0.06127	P=0.8070	F (1, 20) = 1.016	P=0.3256	F (1, 20) = 0.1261	P=0.7262
Hspa1b	F (1, 20) = 8.973	P=0.0071	F (1, 20) = 0.04462	P=0.8348	F (1, 20) = 1.265	P=0.2739
Gdf1	F (1, 20) = 4.5	P=0.0466	F (1, 20) = 0.5537	P=0.4655	F (1, 20) = 0.005	P=0.9443
Hspa1a	F (1, 20) = 1.871	P=0.1865	F (1, 20) = 0.02156	P=0.8847	F (1, 20) = 0.3823	P=0.5434
Doc2g	F (1, 20) = 2.188	P=0.1547	F (1, 20) = 0.03214	P=0.8595	F (1, 20) = 0.1134	P=0.7398
Nr2f2	F (1, 20) = 2.86	P=0.1063	F (1, 20) = 4.145	P=0.0552	F (1, 20) = 0.04434	P=0.8353
Cdhr1	F (1, 20) = 3.383	P=0.0808	F (1, 20) = 0.02546	P=0.8748	F (1, 20) = 0.03637	P=0.8507
Amd1	F (1, 20) = 0.01652	P=0.8990	F (1, 20) = 0.2414	P=0.6285	F (1, 20) = 0.01009	P=0.9210
Ppapdc1a	F (1, 20) = 2.998	P=0.0988	F (1, 20) = 5.561	P=0.0287	F (1, 20) = 0.4394	P=0.5150
Cyp26b1	F (1, 20) = 0.3347	P=0.5694	F (1, 20) = 4.324	P=0.0507	F (1, 20) = 3.538	P=0.0746
Fam181a	F (1, 20) = 0.101	P=0.7539	F (1, 20) = 0.333	P=0.5703	F (1, 20) = 1.312	P=0.2656
Mid1	F (1, 20) = 32.46	P<0.0001	F (1, 20) = 0.2321	P=0.6352	F (1, 20) = 16.57	P=0.0006
Ifit3	F (1, 20) = 2.013	P=0.1714	F (1, 20) = 0.1242	P=0.7282	F (1, 20) = 4	P=0.0593
Atf3	F (1, 20) = 0.1074	P=0.7465	F (1, 20) = 4.987	P=0.0371	F (1, 20) = 0.7883	P=0.3852
Tmem86b	F (1, 20) = 0.08712	P=0.7709	F (1, 20) = 1.147	P=0.2970	F (1, 20) = 0.5348	P=0.4731
Ccdc74a	F (1, 20) = 0.05205	P=0.8219	F (1, 20) = 0.8104	P=0.3787	F (1, 20) = 0.4543	P=0.5080
Akip1	F (1, 20) = 1.164	P=0.2934	F (1, 20) = 1.172	P=0.2919	F (1, 20) = 0.06123	P=0.8071
Oasl2	F (1, 20) = 2.498	P=0.1297	F (1, 20) = 0.08453	P=0.7742	F (1, 20) = 1.754	P=0.2003
Lars2	F (1, 20) = 0.6194	P=0.4405	F (1, 20) = 2.384	P=0.1383	F (1, 20) = 1.139	P=0.2986
Gkn3	F (1, 20) = 1.297	P=0.2681	F (1, 20) = 0.1805	P=0.6754	F (1, 20) = 2.174	P=0.1559

Supplementary Table S3. Two-way ANOVA of background and sex effects on gene expression in wild-type mice.

	Background		Sex		Interaction	
	F (DFn, DFd)	P value	F (DFn, DFd)	P value	F (DFn, DFd)	P value
Mouse tau	F (1, 16) = 0.07445	P=0.7885	F (1, 16) = 0.09461	P=0.7624	F (1, 16) = 3.058	P=0.0995
Gapdh	F (1, 16) = 0.07324	P=0.7901	F (1, 16) = 0.09537	P=0.7614	F (1, 16) = 3.055	P=0.0996
Fgf14	F (1, 16) = 0.6583	P=0.4291	F (1, 16) = 0.2951	P=0.5945	F (1, 16) = 0.1623	P=0.6924
Vipr2	F (1, 16) = 1.428	P=0.2496	F (1, 16) = 0.6472	P=0.4329	F (1, 16) = 0.4998	P=0.4898
Wdr60	F (1, 16) = 0.4475	P=0.5130	F (1, 16) = 0.537	P=0.4743	F (1, 16) = 0.2383	P=0.6320
Esyt2	F (1, 16) = 0.2478	P=0.6254	F (1, 16) = 0.9038	P=0.3559	F (1, 16) = 0.4377	P=0.5177
Ncapg2	F (1, 16) = 0.008313	P=0.9285	F (1, 16) = 0.2447	P=0.6275	F (1, 16) = 0.08555	P=0.7737
D430020J02Rik	F (1, 16) = 0.01459	P=0.9053	F (1, 16) = 0.0002714	P=0.9871	F (1, 16) = 0.2597	P=0.6173
Ptprn2	F (1, 16) = 0.009542	P=0.9234	F (1, 16) = 0.6304	P=0.4388	F (1, 16) = 0.1548	P=0.6992
Cdk5	F (1, 16) = 0.1027	P=0.7528	F (1, 16) = 0.03674	P=0.8504	F (1, 16) = 0.02836	P=0.8684
Dyrk1a	F (1, 16) = 0.9782	P=0.3374	F (1, 16) = 1.039	P=0.3232	F (1, 16) = 0.5456	P=0.4708
Gsk3b	F (1, 16) = 0.064	P=0.8035	F (1, 16) = 4.61	P=0.0475	F (1, 16) = 0.00317	P=0.9558
Csnk1a1	F (1, 16) = 0.1534	P=0.7004	F (1, 16) = 0.06744	P=0.7984	F (1, 16) = 0.04279	P=0.8387
Mark1	F (1, 16) = 0.09484	P=0.7621	F (1, 16) = 0.1678	P=0.6875	F (1, 16) = 0.8114	P=0.3811
Mark2	F (1, 16) = 1.011	P=0.3295	F (1, 16) = 0.9997	P=0.3323	F (1, 16) = 0.6418	P=0.4348
Mark3	F (1, 16) = 0.1043	P=0.7509	F (1, 16) = 0.05765	P=0.8133	F (1, 16) = 0.3069	P=0.5873
Mark4	F (1, 16) = 0.04736	P=0.8305	F (1, 16) = 0.3262	P=0.5758	F (1, 16) = 1.238	P=0.2822
Prkaca	F (1, 16) = 0.2622	P=0.6156	F (1, 16) = 0.1985	P=0.6619	F (1, 16) = 0.1872	P=0.6710
Ppp2ca	F (1, 16) = 0.02417	P=0.8784	F (1, 16) = 0.2741	P=0.6078	F (1, 16) = 0.8634	P=0.3666
Trh	F (1, 16) = 1.803	P=0.1981	F (1, 16) = 0.3222	P=0.5782	F (1, 16) = 2.458	P=0.1365
Hspa1b	F (1, 16) = 1.47	P=0.2430	F (1, 16) = 6.742	P=0.0195	F (1, 16) = 22.7	P=0.0002
Gdf1	F (1, 16) = 0.2911	P=0.5969	F (1, 16) = 0.1688	P=0.6867	F (1, 16) = 1.145	P=0.3005
Hspa1a	F (1, 16) = 0.6461	P=0.4333	F (1, 16) = 4.345	P=0.0535	F (1, 16) = 13.49	P=0.0021
Doc2g	F (1, 16) = 0.1813	P=0.6759	F (1, 16) = 0.1721	P=0.6837	F (1, 16) = 0.09514	P=0.7617
Nr2f2	F (1, 16) = 0.1995	P=0.6611	F (1, 16) = 0.08498	P=0.7744	F (1, 16) = 1.341	P=0.2639
Cdhr1	F (1, 16) = 0.07528	P=0.7873	F (1, 16) = 0.06558	P=0.8011	F (1, 16) = 0.02947	P=0.8659
Amd1	F (1, 16) = 0.6836	P=0.4205	F (1, 16) = 0.3414	P=0.5672	F (1, 16) = 1.143	P=0.3008
Ppapdc1a	F (1, 16) = 3.838	P=0.0678	F (1, 16) = 1.406	P=0.2530	F (1, 16) = 1.651	P=0.2171
Cyp26b1	F (1, 16) = 0.3457	P=0.5648	F (1, 16) = 0.1538	P=0.7001	F (1, 16) = 2.363	P=0.1438
Fam181a	F (1, 16) = 0.02238	P=0.8829	F (1, 16) = 0.4559	P=0.5092	F (1, 16) = 0.01081	P=0.9185
Mid1	F (1, 16) = 7.832	P=0.0129	F (1, 16) = 1.577	P=0.2273	F (1, 16) = 1.235	P=0.2829
Ifit3	F (1, 16) = 0.09585	P=0.7609	F (1, 16) = 0.2107	P=0.6524	F (1, 16) = 0.239	P=0.6315
Atf3	F (1, 16) = 0.3831	P=0.5446	F (1, 16) = 1.787	P=0.1999	F (1, 16) = 1.057	P=0.3192
Tmem86b	F (1, 16) = 0.1316	P=0.7216	F (1, 16) = 0.5241	P=0.4795	F (1, 16) = 1.003	P=0.3316
Ccdc74a	F (1, 16) = 0.4488	P=0.5125	F (1, 16) = 0.3356	P=0.5704	F (1, 16) = 0.8576	P=0.3682
Akip1	F (1, 16) = 0.01828	P=0.8941	F (1, 16) = 0.005804	P=0.9402	F (1, 16) = 0.02385	P=0.8792
Oasl2	F (1, 16) = 1.922	P=0.1846	F (1, 16) = 0.1598	P=0.6946	F (1, 16) = 1.45	P=0.2460
Lars2	F (1, 16) = 0.4452	P=0.5141	F (1, 16) = 0.4827	P=0.4972	F (1, 16) = 1.415	P=0.2515
Gkn3	F (1, 16) = 0.8992	P=0.3571	F (1, 16) = 0.0001368	P=0.9908	F (1, 16) = 0.0002137	P=0.9885

Supplementary Table S4. Top 10 upregulated and downregulated genes in RNA-seq analysis between wild-type_CxF and wild-type_FxC.

Gene	Fold change log_2(CxF/FxC)	Entrez Gene ID	Description
Trh	-1.74	22044	thyrotropin releasing hormone
Hspa1b	-1.47	15511	heat shock protein 1B
Gdf1	-1.46	14559	growth differentiation factor 1
Hspa1a	-1.30	193740	heat shock protein 1A
Doc2g	-1.20	60425	double C2, gamma
Nr2f2	-1.07	11819	nuclear receptor subfamily 2, group F, member 2
Cdhr1	-1.03	170677	cadherin-related family member 1
Amd1	-0.76	11702	S-adenosylmethionine decarboxylase 1
Ppapdc1a	-0.63	381925	phosphatidic acid phosphatase type 2 domain containing 1A
Cyp26b1	-0.60	232174	cytochrome P450, family 26, subfamily b, polypeptide 1
Fam181a	0.82	100504156	family with sequence similarity 181, member A
Mid1	0.99	17318	midline 1
Ifit3	1.01	15959	interferon-induced protein with tetratricopeptide repeats 3
Atf3	1.03	11910	activating transcription factor 3
Tmem86b	1.03	68255	transmembrane protein 86B
Ccdc74a	1.04	72315	coiled-coil domain containing 74A
Akip1	1.14	57373	A kinase (PRKA) interacting protein 1
Oasl2	1.23	23962	2'-5' oligoadenylate synthetase-like 2
Lars2	1.45	102436	leucyl-tRNA synthetase, mitochondrial
Gkn3	1.61	68888	gastrokine 3

Fold change values were calculated as log_2{(FPKM of wild-type_CxF)/(FPKM of wild-type_FxC)}.

Supplementary Table S5. Primer sequences used in qPCR analysis

Target	Forward primer	Reverse primer
Transgenic human tau construct	CCCAATCACTGCCTATACCC	CCACGAGAATGCGAAGGA
Mouse tau exon 7	AGCCCTAAGACTCCTCCA	TGCTGTAGCCGCTTCGTTCT
Akip1	AAGAGTCCTCTTGGCCTACTGA	CAACCACGTGCGTCTTCTTG
Amd1	ATCCCAAGATCTGAATGGGATGTC	GCTTCCTGCTTGTCAGTCTTTG
Atf3	AGTGTGAATGCTGAGCTGAAGG	GTTCCTCTCGTCTTCCGGTG
Ccdc74a	AGATCGAGCACCTGAAGCGG	AGAGTTGGCGGACATCGTG
Cdhr1	AATTGAGGCAACAGACCAGGATG	AACACGTTGACTGGCTCTGC
Cdk5	CCCTGAGATTGTGAAGTCATTCC	CCAATTTCAACTCCCCATTCCT
Csnk1a1	TCCAAGGCCGAATTTATCGTC	ACTTCCTCGCCATTGGTGATG
Cyp26b1	GCAACAAGCGCAAGGTCTTC	TGGATCACCAGTTGGATCTTGG
D430020J02Rik	CAAGTGGTCCTTATGCGGAGG	ATACAGTTCCATTACGGTGTGCT
Doc2g	CTATATGAGGAGGAGGAGATGGAGG	AGCAAGGTGAACACAGCGTAG
Dyrk1a	TGCACCGTCGTTCTCATTCC	CTGGTCACTTATGCTCGGCT
Esyt2	ATGTCAGTCGGTCACAAGGC	GCTCATCTTTAACCTCAACCTCA
Fam181a	TGCTTCTCAGCCTGCACGGA	AGGGCAGACCGAAGGTGAAG
Fgf14	GCGGCTTGATCCGTCAGAA	GAGAAGATATCCACCAGGTTGCC
Gapdh	TGGTGAAGCAGGCATCTGAG	TGCTGTTGAAGTCGCAGGAG
Gdf1	TGCATGTGAGCTTCCGTGAG	CACAGCGTGGTTGAGTGCAG
Gkn3	GCTGACGAACACTAGCGACA	TTGTCTCGGATGCTGACCAC
Gsk3b	CGGAGTTGTCCAGCCAATGA	TAGCGACAGTGGCCAATCAG
Hspa1a	TCGAGGAGGTGGATTAGAGGC	GTCTAGGACTTGATTGCAGGACA
Hspa1b	ACCATCGAGGAGGTGGATTAGA	AACCTTGACAGTAATCGGTGCC
Ifit3	CCTACATAAAGCACCTAGATGGC	ATGTGATAGTAGATCCAGGCGT
Lars2	TGGCCATCTCTCCTAGCCAC	CTGGTGTGAGGCAATCTCTGC
Mark1	TGTGGATGGCTACACTGAAAC	CATCTGTCGCTGACGTAATGG
Mark2	CTACCCACGCTGAACGAAAGG	GTAGTTGCCAATATGGGGCTG
Mark3	AGGCCGAGAGGTTGCAATAA	TCCGTTTCAATGACTTCGAACAA
Mark4	TGGGCAGTGGACGATCTTC	CTTCCCGATGGTCCTTAGCAG
Mid1	AAGGCAAGGTGATCAGGCTC	TTGCGAGATGAGCGATGCAG
Ncapg2	GATCCTTATCCACGGGTTCGT	TCGGCTGAGCTTATATCAAATGC
Nr2f2	AGCAAGTGGAGAAGCTCAAGG	CAGGCATCTGAGGTGAACAGG
Oasl2	TGGAATGTACAGCGAGCGAG	CCTCCTGATCTCTGCCTTCATCT
Ppapdc1a	TGGTGCAGTCAGACAACATACC	AGAGCCAAGGACACTGCTAAGA
Ppp2ca	ATGGACGAGAAGTTGTTCACC	CAGTGACTGGACATCGAACCT
Prkaca	AGATCGTCCTGACCTTTGAGT	GGCAAAACCGAAGTCTGTCAC
Ptprn2	GAGGATGGCTTGTGTGGATCA	CGGAACCTTTTGACATCTTCCAA
Tmem86b	TCTCTGCCTGGTTGTGTTCCTG	CAGAGAAGGCTGCCGTGCCATA
Trh	GAAGGTGCTGTGACTCCTGAC	CCAGGAATCTAAGGCAGCACCA
Vipr2	GACCTGCTACTGCTGGTTG	CAGCTCTGCACATTTTGTCTCT
Wdr60	AGAAGCAAGGTTGGACGGAG	TGACGATGTGAGGCTGTTGC
